# PowerNovo: de novo peptide sequencing via tandem mass spectrometry using an ensemble of transformer and BERT models

**DOI:** 10.1038/s41598-024-65861-0

**Published:** 2024-07-01

**Authors:** Denis V. Petrovskiy, Kirill S. Nikolsky, Liudmila I. Kulikova, Vladimir R. Rudnev, Tatiana V. Butkova, Kristina A. Malsagova, Arthur T. Kopylov, Anna L. Kaysheva

**Affiliations:** Institute of Biomedical Chemistry, 119121 Moscow, Russia

**Keywords:** Bioinformatics, Proteome informatics

## Abstract

The primary objective of analyzing the data obtained in a mass spectrometry-based proteomic experiment is peptide and protein identification, or correct assignment of the tandem mass spectrum to one amino acid sequence. Comparison of empirical fragment spectra with the theoretical predicted one or matching with the collected spectra library are commonly accepted strategies of proteins identification and defining of their amino acid sequences. Although these approaches are widely used and are appreciably efficient for the well-characterized model organisms or measured proteins, they cannot detect novel peptide sequences that have not been previously annotated or are rare. This study presents PowerNovo tool for de novo sequencing of proteins using tandem mass spectra acquired in a variety of types of mass analyzers and different fragmentation techniques. PowerNovo involves an ensemble of models for peptide sequencing: model for detecting regularities in tandem mass spectra, precursors, and fragment ions and a natural language processing model, which has a function of peptide sequence quality assessment and helps with reconstruction of noisy sequences. The results of testing showed that the performance of PowerNovo is comparable and even better than widely utilized PointNovo, DeepNovo, Casanovo, and Novor packages. Also, PowerNovo provides complete cycle of processing (pipeline) of mass spectrometry data and, along with predicting the peptide sequence, involves the peptide assembly and protein inference blocks.

## Introduction

Tandem mass spectrometry allows rapid and accurate detection of peptides and proteins in complex biological samples. However, protein identification in the mass spectrum still remains a challenge. The main difficulty consists in spectrum identification according to mass and charge of a precursor ion and detected fragments. Our objective is to identify the amino acid sequence of the precursor ion corresponding to the original peptide sequence.

Database search was performed by systematically checking all the possible candidate peptides that can correspond to the observed spectrum. Matching of the spectrum with each peptide in the list of candidates is evaluated, and the peptide characterized by the best match is chosen (PSM—the peptide spectrum match). However, a drawback of this approach is that one needs to know in advance what peptides the sample may contain. This approach also has a number of other significant shortcomings. First, it requires a large number of computational resources and time to process each spectrum. Second, unexpected peptide sequence variants resulting from genetic variations or modifications can be missed. Third, this method cannot be used to analyze samples for which no comprehensive information about genome sequence is available (e.g., when analyzing the microbiome or the immunopeptidome).

Our study overviews the results of using the most common de novo sequencing tools (Table 1), including those employed to conduct a comparative analysis of performance for the proposed solution and the currently known algorithms.

**Table 1:** An overview of de novo peptide sequencing tools.

Software tool	Algorithms and methods	Year	References
Novor	Spectrum graph Decision Tree	2015	^1^
DeepNovo	CNN for spectrum peak embedding CNN for spectrum processing RNN for peptide sequence prediction Dynamic programming for post-processing Discretization of m/z axis	2017	^2^
SMSNet	CNN for spectrum peak embedding CNN for spectrum processing Database search post-processor (peptide patterns search)	2019	^3^
PointNovo	CNN for spectrum processing. (based of PointNet model) RNN for peptide sequence prediction Dynamic programming for post-processing	2021	^4^
Casanovo	Transformer-based deep learning model	2022	^5^

The name, algorithms used, year of publication and the corresponding reference are displayed for each tool.

The de novo sequencing algorithms are evolving in tandem with mass spectrometry technology in line of higher accuracy and performance. Early algorithms were based on spectrum graph and decision tree machine learning methods (Novor; Table 1). The most obvious disadvantage of the spectrum graph approach is relatively low performance, which becomes even worse the higher spectra resolution you acquire^6^. Recently, neural network with different architectures are becoming deeply involved in sequencing algorithms what significantly increases the performance of de novo sequencing^7^. DeepNovo, PointNovo, and SMSNet realize the architecture of deep neural network with consequent post-processing to definite pinpoint misidentified residues. The evolution of neural network architectures follows from convolution neural network (CNN), recurrent neural network (RNN) и long short-term memory (LSTM), which were being popular in DeepNovo, PointNovo and SMSNet, to currently used Transformer which is successfully incorporated in Casanovo. All these methods are characterized by better performance compared to the early elaborated ones but still bear some disadvantages.

The recent reports involving the data on comparative analysis of the available de novo solutions demonstrate that these methods ensure a 39–60% peptide-level recall depending on the initial dataset^4,8,9^. The deep learning models with transformer-based framework ensure better performance based on the models having other architectures (RNN, LSTM, CNN).

The following main problems of de novo peptide sequences can be identified based on the analysis:

1. Generation of noisy peptide sequences with incorrect amino acid positions^8–10^.
2. The absence of hydrolysis sites and the presence of noise abruptly reduces the algorithm accuracy and is a source of prediction errors. Noise in the spectra often causes false positive predictions^9^.
3. Prediction accuracy is inversely proportional to length of the amino acid sequence of peptide.
4. The total coverage of long protein chains (> 350 AA) is ≤ 20%.

This problem is typically solved using additional post-processing stages that involve either comparison of the predicted peptide mass to the precursor mass observed in the spectrum using dynamic programming or prediction refinement of the models by searching for characteristic peptides across the database.

There is another problem intrinsic to all the deep learning models at the sequence decoding stage. All the models usually predict the peptide sequence iteratively: from the first token to the last one (sometimes in inverse order), and correctness of next token prediction directly depends on whether the previous one was predicted properly. This often results in wrong predictions because of errors made at early decoding stages. Although this problem is characteristic of all the language models, it is most pronounced during spectrum decoding because of the peptide fragmentation features. The first products of peptide fragmentation are often not observed in the spectrum owing to the first mass cutoff during spectrum recording; their signals can also be highly noisy because of their physicochemical properties. This problem is solved using the beam search strategy. In this strategy, we do not immediately select the next token at each step, but rather memorize several variants of the most probable tokens (hypotheses). It is typically done using the matrix whose width equals to sequence length and height equals to the number of symbol generation variants (beam search width). Once generation of sequence variants is completed, an algorithm for variant assessment is used to select the most probable sequence. The beam search strategy is one of the most common methods among de novo tools (Table 1) and is successfully used to solve problems with short sequences; however, this problem becomes increasingly noticeable at longer lengths. Furthermore, this strategy has another shortcoming: for the hypothesis basket of limited size (5–10 hypotheses), the correct hypothesis can be replaced from the basket with an incorrect one if the model assesses its confidence score to be higher. There currently is no single well-proved algorithm for assessing sequence variants; they are mostly assessed according to the confidence score of the model.

This study offers a de novo peptide sequencing tool based on an ensemble of models. The first model, a transformer, translates the observed spectral peaks of variable length to the peptide sequence. The second model, BERT (Bidirectional Encoder Representations from Transformers), assess the peptide sequences generated by the first model.

The BERT model has been trained using a large array of peptides and performs the following functions:

- predicts peptide detectability in proteomics studies;
- predicts that the peptide sequence under consideration does not exist in nature (decoy sequences);
- corrects amino acid residues in noisy peptide sequences.

The BERT model is closely integrated with the beam search algorithm and is used to evaluate the variants of generated sequences. Due to this tool, we have solved some problems related to noisy sequences: got rid of deliberately false predictions at early stages, thus improving quality of the results and solution performance.

The experiments for comparing the performance to that of other common de novo sequencing tools were conducted using monoclonal antibody datasets. The results of the experiments demonstrated that the solution reported in this paper predicts peptide sequences with a noticeably higher precision compared to other modern methods. Furthermore, the number of peptide and protein identification errors was noticeably smaller.

It should be mentioned separately that the reported solution performs a complete cycle of processing (pipeline) of mass spectrometry data and, along with predicting the peptide sequence, involves the peptide assembly and protein inference blocks.

## Materials and methods

### Overview of datasets

The models were trained using large training datasets containing spectra for various species, such as *Homo sapiens*, *Mus musculus*, *Escherichia coli* and Yeast. The total number of peptides in the training set exceeds 3.5 million. The BERT model was trained using 4.5 million peptide sequences retrieved from such sources as MassIVE-KB, GPMDB^11^, and Peptide Atlas^12^ and 16 million decoy sequences obtained using different methods: shuffle, reverse, shuffle with and without conservation of cleavage sites.

#### Annotated spectrum datasets

The Transformer model for translating spectra to peptide sequence was trained using annotated spectra of MassIVE-KB database^13^ and NIST spectral library^14^. To test the performance of Transformer model we collected spectra data of *Homo sapiens*, *Mus musculus*, *Escherichia coli* and Yeast, acquired by different LC–MS/MS with various types of fragmentation, including HCD, CID, and ETD fragmentations, that produce a-, b-, c-, y-, and z-types of fragment ions. The majority of spectra were generated in data-dependent mode (DDA), while the data-independent analysis (DIA) comprised dramatically less fraction of testing dataset. The description of training dataset and description of dataset admitted for evaluation of performance are available in Supplementary Tables S1 and S2. To test the performance of antibodies sequencing, we selected human IgG1 and Herceptin datasets (Supplementary Table S4).

#### Datasets for training peptide detectability

The dataset for training was obtained by merging two datasets: GPMDB and Massive-KB. Massive-KB contains 2,492,495 peptides obtained from 24,677 proteins. After pre-check, the list was limited to 16,051 proteins with ≥ 50% sequence coverage. Next, after virtual trypsin cleavage of proteins and assessment of frequency (3^rd^ quantile of the group distribution), we selected 94,271 unique peptides that were classified as highly detectable. The remaining peptides were classified as potentially detectable.

In the GPMDB dataset comprising 1,839,011 peptides, we also identified peptides whose frequency was in the 3rd quantile of the group distribution (n = 81,016). The remaining 1,558,995 peptides were classified as potentially detectable.

After merging the datasets and removing duplicates, we ended up with 163,053 highly detectable peptides and 4,268,960 potentially detectable peptides.

Separately, we created four decoy datasets according to the Peptide Atlas data (Human all proteome, THISP_2024-01-01) using the Galaxy Proteomics Lab service^15^. Decoy datasets were generated using the technology of sequence shuffling and reverse translation; positions of cleavage sites were conserved in two datasets. The total amount of sequences in the decoy datasets was 16,267,014.

Supplementary Table S3 lists the characteristics of the dataset used to train the BERT model. The dataset was highly imbalanced, and we used the procedures of dataset partitioning, weighted sampling, and proportionally increasing class weights for training the model.

Datasets for training and validate models are available in the MassIVE repository with Library Name: KB 2.0.15, in vivo^13^, dataset identifier MSV000081142, Peptide Atlas data with Library Name: THISP_2024-01-01^16^ and GPMDB (https://gpmdb.thegpm.org/).

### Overview of architecture

PowerNovo objectifies complete cycle of mass spectrometry data processing. Its architecture connects several modules, each of one commits a very specific function. The architecture can be subdivided for several functional blocks:

1. The ‘Transformer model’ module is highly capable to learn contextualized representations of input data and modeling sequences. In the PowerNovo architecture, the Transformer model works under the problem of sequence-to-sequence learning, where sequences of the observed spectral peaks of variable length are translated into sequences of amino acids of variable length. In general, this module is responsible for processing of spectra, learning their latent representations and predicting peptide sequences.

The ‘Transformer model’ comprised of an encoder, which is responsible for spectra processing, features extraction, and generation of hidden spectra representation; and a decoder, which accomplishes the transformation of hidden spectra representation into amino acids sequences. The decoder is integrated in Beam search, which is a method of decoding a sequence using an autoregressive function that outputs a probability distribution over the next possible symbols. Ideally, the search algorithm should go through all the paths and select the most likely sequence hypothesis. However, this is prohibitively expensive. Typically, the size of the considered hypotheses (beam size) does not exceed 5. In our architecture, we use the BERT neural network model to evaluate peptide sequence hypotheses.

2. Peptide inference module. In mass spectrometry experiments, it is usually possible to observe only a part of the theoretical peptides, hampering characterization of proteins. Thus, the detectability of peptide can be defined as the probability to detect a peptide in a standard sample using a standard proteomic procedure, and it serves as an indicator for evaluating the quality of identified results. The mechanism of peptide detection is complex due to efficiency of enzymatic cleavage, ionization efficiency, post-translational modifications, etc.^17^ Therefore, researchers increasingly turn to a computational approach to predict the probability of detecting peptides accurately. To date, various studies have been conducted to characterize the mechanism of peptide detection and achieve accurate prediction of peptide detectability using computational methods^18–20^ but, recently, researchers frequently choose rather machine learning^21,22^ to solve this problem.

The PowerNovo exploits a model based on the BERT architecture, which is responsible evaluation of peptides under the interest, correction of amino acid residues and prediction of peptides detectability. The input of this module includes hypotheses of amino acid sequences from the output of the Transformer. The amino acid sequences of the hypotheses are evaluated, corrected if necessary, and each hypothesis is assessed by its detectability as decoy, highly detectable or poorly detectable. The result of the module is the most probable peptide sequence for each spectrum and its confidence score.

**Figure 1:**
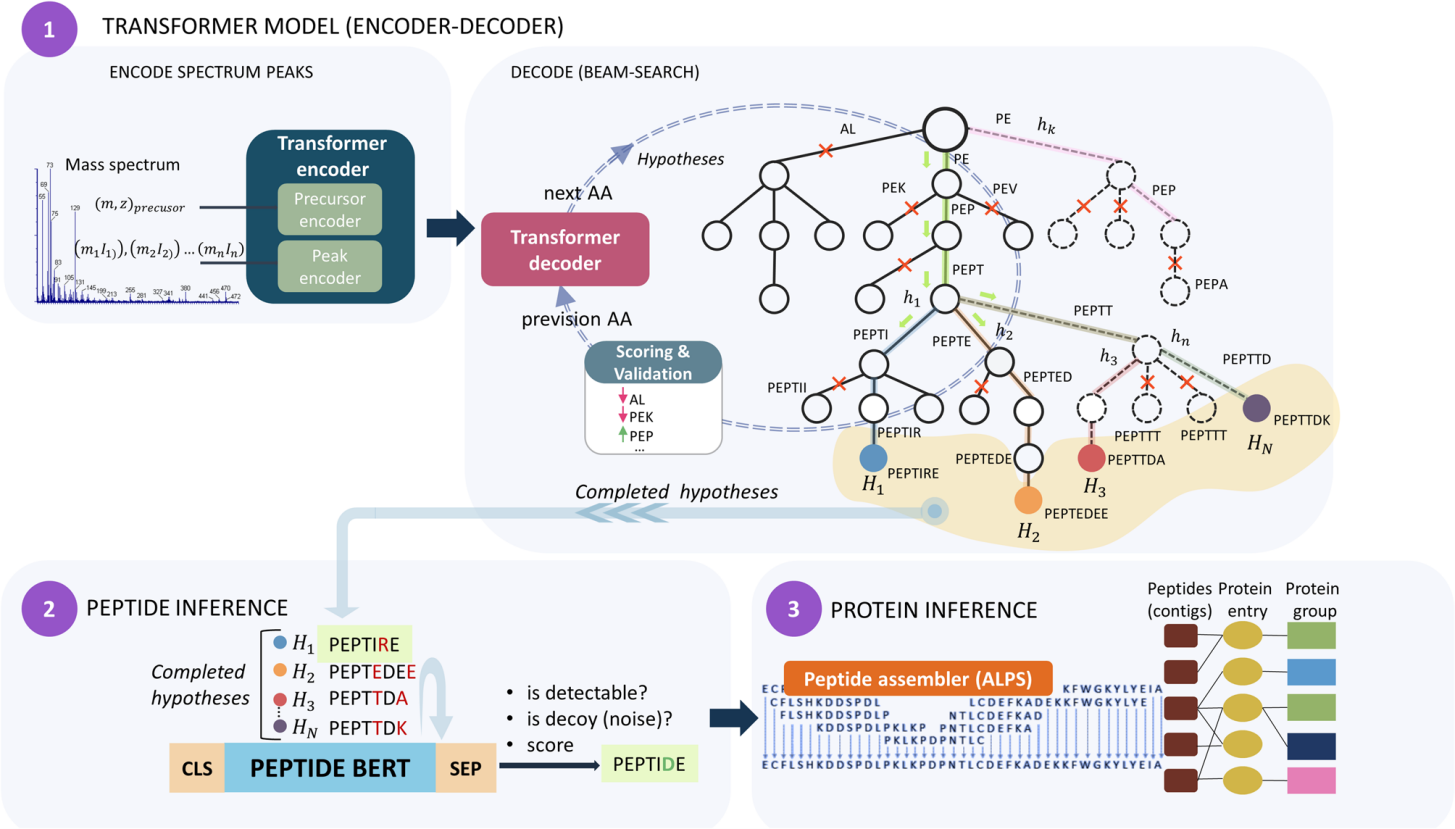
PowerNovo architecture overview. (1) The model takes the peak mass spectrum (m/z and intensity) as input data, which is transformed to embedding using sinusoidal encoder. This is passed through transformer encoder layers into a hidden representation. Information about the precursor is also encoded and connected to the hidden representation of the spectrum. The decoder takes the embedding from the encoder and iteratively predicts the next amino acid in the peptide. Decoding is performed using a beam search strategy to ensure that the model outputs a confident prediction that matches the precursor mass and charge as much as possible. (2) Peptide Inference. PowerNovo includes a BERT model, whose primary function is to evaluate the sequence hypotheses formed at the output of step 2 and to output the most suitable one. (3) The protein inference module is responsible for assembling peptides into contigs, matching them with target proteins and outputting target.

3. Protein Inference module. Protein inference is one of the important steps in protein identification, which transforms peptides identified from mass spectra into a list of proteins. The transformation of peptide sequences into protein is accomplished by three steps (Fig. 1). On the first step peptides are assembled in contigs as a reconstructed sequences from sequence alignment of reads, which give rise to a long protein sequence. The transformation occurs bolstering by the De Bruijn ALPS sequence assembler^23^ and outputs a file with a set of contigs. On the seconds step, the resulting set of contigs is used to detect and identify proteins through customized FASTA database, utilizing high-throughput ‘npyseach’^24^ library, which realizes search and alignment functions as that known in BLAST algorithm. A file containing contigs and corresponding identified proteins is outputted. The fords step is Protein Inference, which gathers and groups all the results into proteins in support of algorithm described in subsection “2.2.4 Protein Inference”.

### Implementation details

#### Transformer model

The model responsible for translation of a spectrum to a peptide sequence is based on the conventional Transformer framework consisting of encoder and decoder blocks^25^. Encoder processes spectral peaks where each peak is characterized by the m/z value and the monotonically related intensity $S=\{({m}_{i}, {I}_{i}){\}}_{i=1}^{N}$. Recent studies have demonstrated that mass spectrometry vectors can be better represented using sinusoidal embeddings^26^. In this case, the m/z peaks are encoded with different frequencies along the hidden dimension of encoded output signal. We used this approach, and the m/z values in our solution were mapped as d-dimensional vectors *f* using the sinusoidal positional encoder (1). The spectrum embedding consists of an equal number of sine and cosine waveforms spanning the wavelengths from 0.001 to 10,000 m/z.

1 $${f}_{i}= \left\{\begin{array}{c}\text{sin}(\frac{{m}_{i}}{\frac{{\lambda }_{max}}{{\lambda }_{min}}(\frac{{\lambda }_{max}}{2\pi }{)}^\frac{2i}{d} }), for \;i \le \frac{d}{2}\\ \text{c}\mathit{os}(\frac{{m}_{i}}{\frac{{\lambda }_{max}}{{\lambda }_{min}}(\frac{{\lambda }_{max}}{2\pi }{)}^\frac{2i}{d} }), for\; i> \frac{d}{2}\end{array}\right.$$

where *λ*_*max*_ = 10,000 and *λ*_*min*_ = 0.001. This mapping ensures encoding of the m/z position by analogy with encoding of word position in language models. The intensity values, which are measured with lower precision in mass spectrometry experiments than the m/z values, are mapped as d-dimensional vectors through the linear layer of the neural network; the m/z embeddings and intensity embeddings are then summed to obtain the input spectral embedding.

The decoder generates a peptide sequence from hidden variables of the encoder in the auto-regressive form (predicts the next token based on previous predictions). We also combine the information provided by hidden variables on precursor weight, m/z, and charge; this information is also valuable for prediction.

This simple but powerful architecture, which actually is a sequence2sequence model, reliably predicts the next tokens of peptide sequences based on mass spectra.

The decoder block is integrated with the beam search strategy based on the knapsack optimization algorithm. It guarantees that the model always retrieves the peptide sequence matching the m/z value of the precursor as much as possible. The algorithm is also integrated with the BERT network evaluating the sequences:

- if the model evaluates the generated peptide sequence as a decoy sequence, it is excluded from the search space;
- sequences evaluated as highly detectable are characterized by top priority among other variants;
- elements of amino acid residues are masked and passed through the BERT model. The possible amino acid residues predicted by the model are also taken into consideration when the final prediction is made.

Therefore, there is no need in multiple predictions; the search space is reduced and model reliability is simultaneously increased, thus decreasing the FDR. Although it imposes additional computational costs and requires more iterations, this approach is justified as it mitigates almost all types of errors.

The detailed overview of the architecture is shown in Supplementary Figs. S1, S2.

#### Peptide BERT model architecture (assessing the quality of peptide sequences and eliminating noise)

In our solution, the BERT model is used to evaluate peptide sequence variants generated by the model described previously. This model is based on the conventional BERT architecture^27^ and is a neural network consisting of compositions of transformer encoders. We used the pre-trained ‘lightweight’ model DistilProtBert^28^ fine-tuned to work with short peptide sequences as a basis, which is called Peptide BERT in the PowerNovo (Supplementary Fig. S2). The model was trained on a large dataset of peptides and decoy sequences using the “masked language model”, which randomly selects 7% of amino acids from the peptide sequence thereafter substitutes 50% of the selected amino acids by new randomly selected amino acids, whereas the rest 50% are replaced with a special mask symbol ([MASK] token). During training, the network must restore the original sequence. The model also predicts the class of peptide sequence (0…2), where 0—is decoy; 1—is highly detectable; and 2—is poorly detectable (or potential detectable).

#### Peptide assembler

PowerNovo involves the stage of reconstructing short peptide sequences derived from network predictions into long sequences. This allows us to make comparisons with proteins and characterize them.

PowerNovo is integrated with de Bruijn ALPS 17 sequence assembler^23^. According to the authors’ description, the k-mer dimension equal to 7 ensures sufficiently high coverage of the amino acid sequence and simultaneously prevents repetition of the resulting contigs. ALPS also takes the confidence score of the network into consideration during sequence assembly, but it may yield incorrect results when a large number of k-mers with low confidence score are used. In our solution, k-mers with k = 7 are used by default.

#### Protein inference

Peptide identification is only an intermediate stage; researchers are usually interested in conclusions whether a sample contains proteins. Our solution presents a module responsible for searching for peptides across the database specified by the user (FASTA file).

However, protein identification according to peptide sequences is also a problem known as the “protein inference problem”. Its key factors are that there are “one-hit wonder” and degenerate peptides among the results of the experiment. The problem of “one-hit wonders” refers to proteins containing a single identified peptide. Since the modern peptide identification algorithms are still far from being perfect, the identified peptide can be false-positive regardless of its uniqueness. The only evidence supporting that this peptide is present is not reliable even if this protein is unique across the entire protein database. Degenerate peptides are those that are shared by several proteins. Compared to unique peptides that are present in a single protein, degenerate peptides cause problems, since it is difficult to understand what protein or group of proteins they refer to. To a great extent, these problems are solved by using a sequence assembler described in the previous section, but the problem still remains. In our solution, we have supplemented the final stage with an algorithm for peptide sequence assembly into proteins, which is simple but appreciably efficient.

The Pipeline involves the following stages:

- After sequence of peptides is assembled by the assembler, we find proteins matching them using search algorithm provided by npyseach library^24^. Algorithm is based on the idea that a few good candidate sequences are sufficient for most sequence similarity searches. Candidate sequences are selected from a database based on the number of unique words or k-mers (the collection of which is designated U) they share and are checked in decreasing order of U for their sequence similarity. If the similarity exceeds a user-defined threshold, the candidate is considered a hit. If the similarity is below the threshold, the candidate is considered a reject and the next best candidate is checked, until the maximum number of allowed rejects for this query has been reached. The performance of the algorithm is comparable to the well-known algorithms for searching and aligning sequences BLAST + ^29^ and VSEARCH^30^.
- Next, we conduct iterative scoring of proteins and associated peptide contigs and cluster them into proteins according to the following algorithm:
- Unscored proteins are assigned scores equal to the sum of associated peptide contigs scores (confidence score from model output).
- Protein with the highest provisional score is assigned the peptide contigs currently associated with it. These contigs are excluded from further consideration.
- If any protein now lacks any possible peptide contigs that are unassigned, it is assigned a "subset" protein which was last assigned peptides.
- Repeat steps a, b, c until all contigs are assigned and all proteins are scored.

Therefore, the Pipeline starts with searching for proteins corresponding to contigs assembled by the assembler and then clusters contigs into proteins based on their scores and associations.

The output files are tables containing contigs and associated proteins/groups of proteins suitable for further analysis. The strategy is encouraged by the adapted REPRISAL algorithm (Recursive Protein Inference Scoring Algorithm), which is used to run Mass Dynamics^31^.

## Results

We have elaborated the PowerNovo innovative solution ensuring the complete cycle of processing mass spectrometry data, starting from translation of peaks to peptide sequences and ending with assembling the peptide “cocktail” into full-length proteins. The model was trained using one of the largest sets annotated proteomic data from different experiments and species. In this section, we analyze the performance of the model, compare it to other state-of-the-art de novo software packages.

### Evaluation

We evaluated the performance of our PowerNovo in two experiments. In the first experiment we used different sets of spectra (different species and fragmentation types). In the second experiment, we evaluated performance on the antibody sequencing task. The analysis involves comparison to state-of-the-art de novo software packages (Table 1), studying the common types of errors as well as analyzing the effect of noisy spectra and the absence of fragmented ions on prediction output.

#### Experiment 1: various sets of spectra

In this experiment we elucidate the performance of PowerNovo by using a large annotated dataset of *H. Sapiens*, *H. Sapiens* with phosphorylation, *H. Sapiens* (noncryptic), *H. Sapiens* (hair), *M. Musculus*, *E. Coli*, and Yeast. Spectra are differed by detector and mass analyzer types (Ion Trap, Orbitrap) and by types of collision dissociation (CID, HCD, ETD). Precision and recall calculated at the amino acid and peptide levels we chosen as an established performance measure. For each spectrum we compare the predicted sequence to the ground truth peptide. In the evaluation, we assumed that a predicted amino acid was considered a “match” with a real amino acid if their masses differed by less than 0.1 Da and if the masses of the prefixes before them differed by less than 0.5 Da^2^. Such an approximate match is used instead of an exact match because of the resolution of the benchmark datasets. We calculated the total recall (and precision) of de novo sequencing as the ratio of the total number of matched amino acids over the total length of real peptide sequences (and predicted peptide sequences, respectively) in the validation dataset. For peptide predictions, a predicted peptide is considered a correct match if all of its amino acids are matched. To plot a precision-recall curve, we sort predictions by the confidence score provided by the model.

##### Test datasets

The analysis was performed for datasets obtained from the NIST and MassIVE-KB proteomics data repositories. This data set combines a total of about 120,000 samples of mass spectra from thirteen different experiments. The dataset contains sets of spectra for three species (*H. sapiens*, *M.musculus*, and Yeast), covering different types of collision dissociation (HCD, ETD, and CID). Based on the identification against the database, each spectrum has already an assigned peptide sequence, which is considered as the ground truth for the evaluation of methods (Supplementary Table S2).

##### Evaluation results

**Figure 2:**
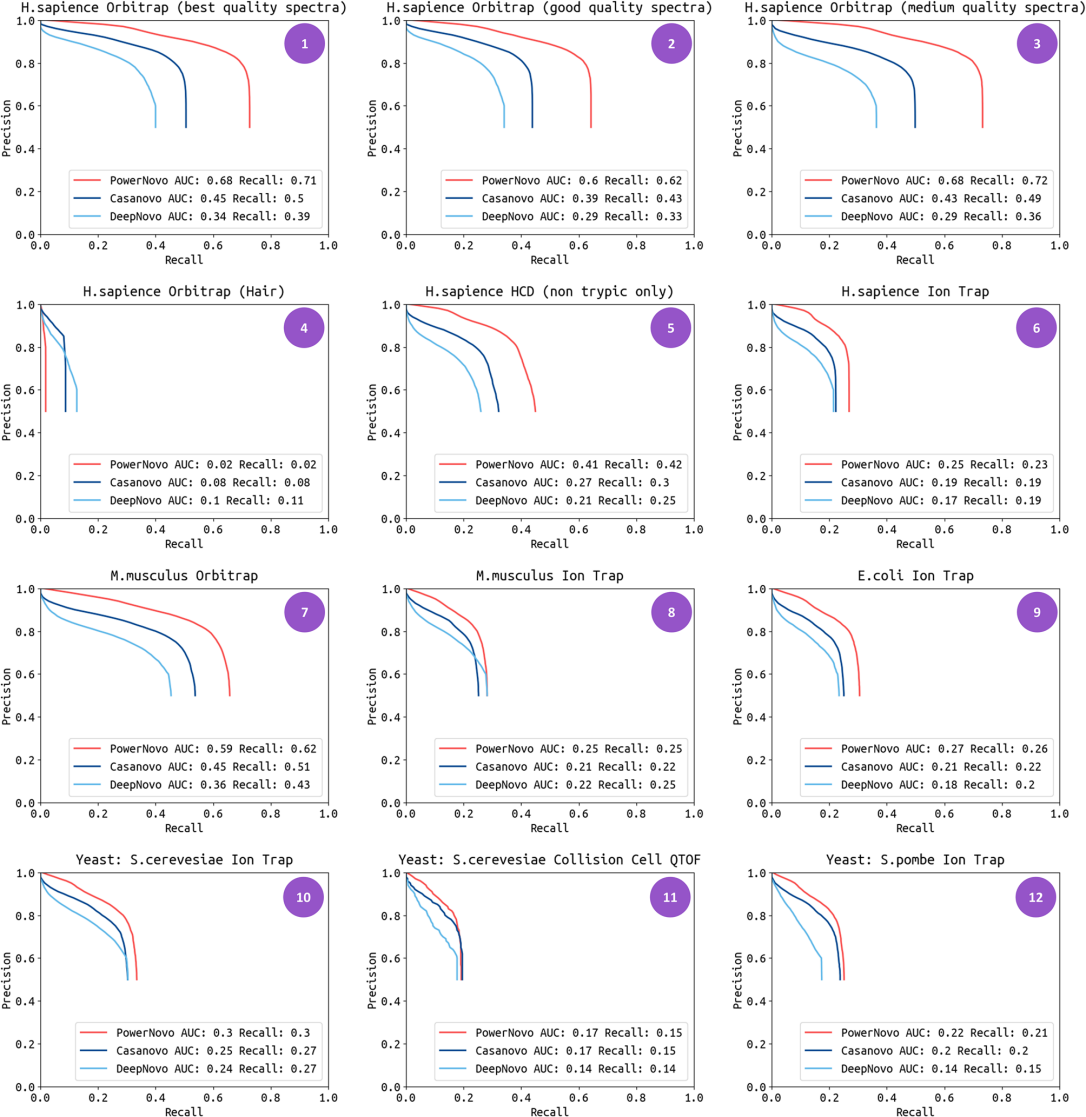
Precision-recall curves for PowerNovo, Casanovo and DeepNovo. Curves are shown for each species at the peptide level. Curves are computed by sorting predicted peptides according to their confidence scores. Descriptions of the de novo testing datasets are provided in Supplementary Table S2. (1) H. sapiens. High-quality HCD spectra, mostly tryptic peptides without missed cleavages. (2) H. sapiens. Medium-quality HCD spectra, mostly tryptic peptides with missed cleavages. (3) H. sapiens. High and medium quality HCD spectra, mostly semi-tryptic peptides (NIST). (4) H. sapiens. Human Hair Peptide HCD Spectral Library (5) H.sapiens. HCD Spectra of peptides from proteomics experiments digested with various different enzymes. Only non-tryptic peptides. (6) H. sapiens. Human Ion Trap Library. (7) M. musculus. Mouse HCD Library. (8) M. musculus. Mouse Ion Trap Library. (9) E.coli. E.coli Ion trap library. (10) Yeast: S. cerevesiae. Yeast Ion Trap Library. (11) Yeast: S. cerevesiae. Yeast Collision Cell (QTOF) Library. (12) Yeast: S. pombe. Yeast Pombe Ion Trap Library.

We evaluated the performance of PowerNovo compared to two state-of-the-art neural network-based methods, Casanovo and DeepNovo. To characterize the performance of Casanovo and DeepNovo we used publicly available pretrained model weights^32,33^. In this comparison, peptide-level accuracy metrics are the primary quantifier of the sequencing model’s practical utility, since the goal is to assign a complete peptide sequence to each observed spectrum (Fig. 2).

PowerNovo demonstrates higher performance (Fig. 2) with an average improvement of recall up to 10% and 14% compared to Casanovo and DeepNovo, respectively (details of recall and precision on the level of peptides are available in Supplementary Table S6). Specifically, the most improved precision is monitored for human proteome spectra due to, apparently, the prevalence of human proteome spectra compared to other species (49% of spectra belong to *H.sapiens*, 20% of *M musculus*, and only 3% of spectra belong to *R. norvegicus* according to the Proteome Xchange metrics). It should be noticed that high performance is representative for all participants of *H. Sapiens* collection apart from *H. Sapiens* (hair) dataset, which was downloaded from the NIST (Table S2. Description of the test datasets). Neither of tested de novo sequencing packages returned a satisfied results for the *H. Sapiens* (hair) dataset, which is assumingly caused by the grade of spectra library or sample preparation quality, or by distinctive data distribution within this dataset. It is expected that the performance of CID spectra is worse grade than of HCD spectra. Probably because the HCD technique produces better fragmentations. Similarly, PowerNovo demonstrates higher improvement compared to Casanovo and DeepNovo in the level of amino acid residues (Supplementary Table S7).

Precision-recall of PTMs detectability in peptides such as phosphoserine, *O*-phosphotyrosine, phosphothreonine is shown separately (Supplementary Fig. S4). The precision-recall indicators of PTMs identification are available for PowerNovo solely because other tested packages for de novo sequencing support canonical (unmodified) amino acid sequences only. Because the number of perfectly or well-annotated PTM spectra with high resolution is relatively low, the recall value makes only 0.21. Currently, we are working hard to collect a satisfied library of PTM spectra to fine tune the model.

The analysis shows that PowerNovo meets the highest requirements. Its performance is comparable or even favors to other tools (Casanovo and DeepNovo), and the model is widely applicable for various types of spectra in irrespective of collision dissociation type, mass analyzer type, and biological species.

#### Experiment 2: antibody sequencing

Confirmation of the amino acid sequence and obtaining information about the sequences of antibodies (Abs) play a crucial role for understanding the structural foundations of binding, recognition and interactions of antigen–antibody complexes. Most sequence diversity is concentrated in hypervariable loops within the antibody variable regions known as the complementarity-determining regions (CDRs), which are generally responsible for the interaction between the antibody and its targets. Antibodies are often of poor quality, which results in poor reproducibility of analytical data in the study. The problem related to antibody classification and identification is still rather relevant, often leading to the so-called replication crisis^34–36^. Because of the presence of variable regions, antibody sequencing is quite a challenging task, which was the key reason why antibodies were selected to be the study object for evaluating the performance of PowerNovo and comparing it to other modern solutions.

As part of performance analysis, we reproduced the experiment described by Beslic et al.^8^ and augmented it with PowerNovo metrics. We also extended some of the conclusions drawn in the original article to better present the results of our study.

##### Antibody datasets

The analysis was performed for three open-source antibody datasets from the MS PRIDE and MassIVE-KB proteomics data repositories. We analyzed the mass spectrometry proteomics data from the proteomics MS data repositories MassIVE and PRIDE with the dataset identifiers MSV000079801 and PXD023419 respectively. The complete parameters of the datasets used are presented in Supplementary Table S4.

##### Parameters for launching de novo software packages

In order to keep our analysis related to the study by D. Beslic et al., we left the run settings of the de novo software package unchanged and adapted some parameters of our PowerNovo solution according to those reported in Ref.^8^. We also used weights of the deep learning models reported in this paper (it applies to the DL DeepNovo, PointNovo, SMSNet, and Casanovo models). All the models were trained on the MassIVE-KB HCD library dataset containing 1,114,503 peptides^37^. Hyperparameters for running de novo packages are described in Supplementary Table S5.

Although all the run settings of the de novo packages were borrowed from the study by Beslic et al.^8^ describing this experiment, the results presented in our paper can slightly differ from the original experiment. We attribute it to the stochastic nature of neural networks as well as rounding errors during calculations and result output.

#### Evaluation results

##### Performance at the peptide and amino acid level

The overall performance of the de novo algorithms was evaluated for six variants of enzymatic cleavage of IgG1-Human-Heavy Chain (Hc). Figure 4 shows the diagrams depicting the performance of de novo algorithms at the amino acid and peptide levels. All the predictions made by the algorithms, regardless of their confidence score, were used to build the diagrams. A number of conclusions can be drawn by analyzing these data. In the case of hydrolysis catalyzed by proteinase-K and Glu-C, the SMSNet and PointNovo algorithms ensured the good precision at the amino acid level, like the one achieved in the original experiment by D. Beslic et al. PowerNovo showed no significant results for these enzymes. For all other enzymes, the performance was better by 3–10% compared to the best results achieved using the DL Casanovo model, which was the leader in the original experiment. Both the Casanovo and PowerNovo models are based on the transformer deep learning framework, so we attempted to find the underlying reason for that. According to our studies, this gain in performance was primarily due to the dimensionality of the networks (nine encoder/decoder layers in Casanovo and ten in PowerNovo), as well as differences in data post-processing. The presence of the BERT block in PowerNovo has no substantial effect on performance at the amino acid level.

**Figure 3:**
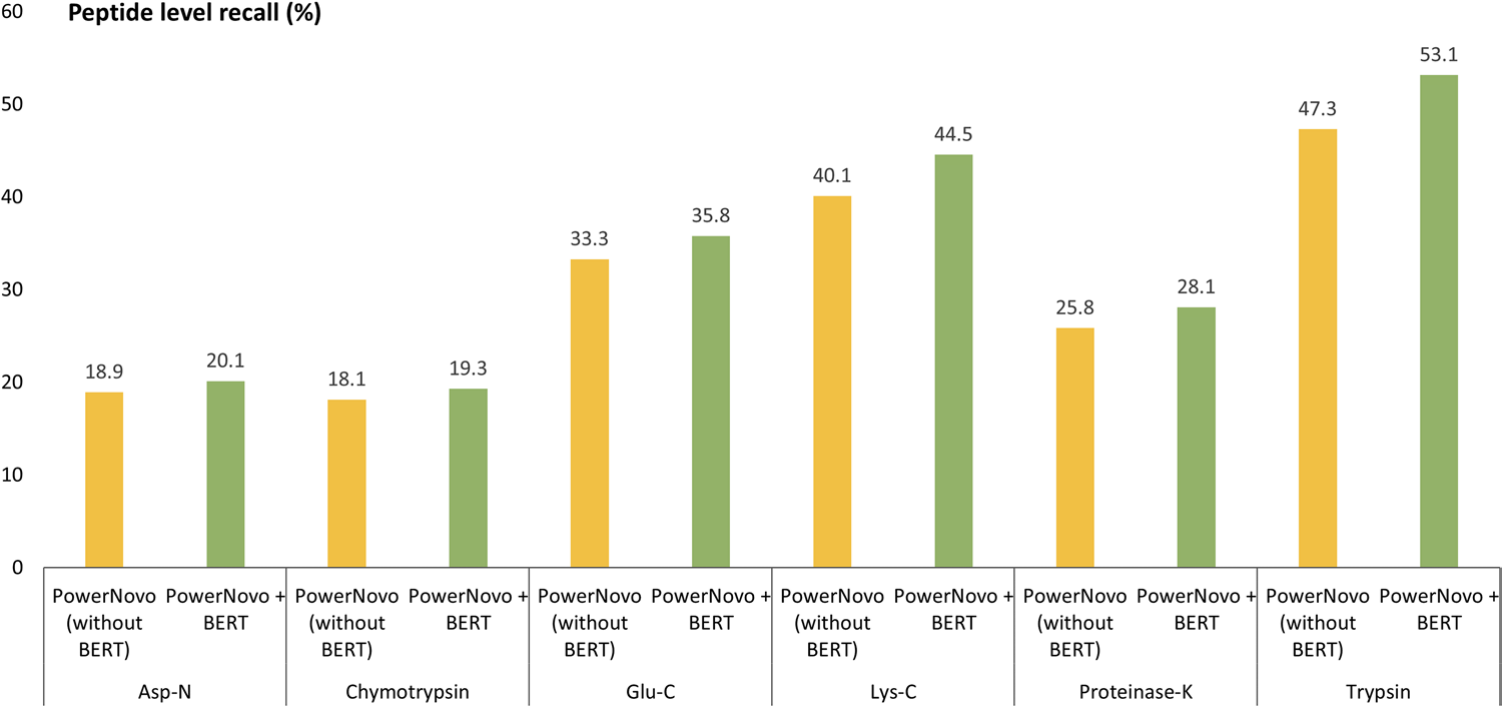
Comparing the performance of the PowerNovo model with and without the BERT module. Recall at peptide level across different enzymes on IgG1-Human-HC (dataset MSV000079801).

Significant gain is observed for peptide level precision. The PowerNovo model made the greatest number of correct peptide predictions compared to other de novo algorithms for all six enzymes. Two aspects can be emphasized here. Prediction accuracy is directly related to the presence of a transformer-based framework: it is also seen in the outputs of the DL Casanovo model. Furthermore, the precision of peptide prediction was found to be significantly affected by the BERT block in PowerNovo. The presence of this block prioritizes prediction of complete highly detectable peptide sequences over partially correct and noisy sequences. Moreover, hypothesis evaluation using the BERT block reduces the number of false positive peptide sequences, whereas in the absence of the BERT block, peptide level recall decreases by 5–15% (Fig. 3).

Setting different thresholds for confidence score algorithms allows us to obtain different sets of predicted peptides. At a sufficiently high threshold, we will end up with a small number of peptides with high precision, but a significant part of the dataset will be excluded, which will reduce the recall and coverage of the target protein by peptides. Meanwhile, setting a low threshold will increase the number of false positive and noisy peptide sequences. Supplementary Fig. S3 shows the precision–recall (PR) curve at the amino acid sequence level for different threshold values (0–95%, with a 5% increment).

**Figure 4:**
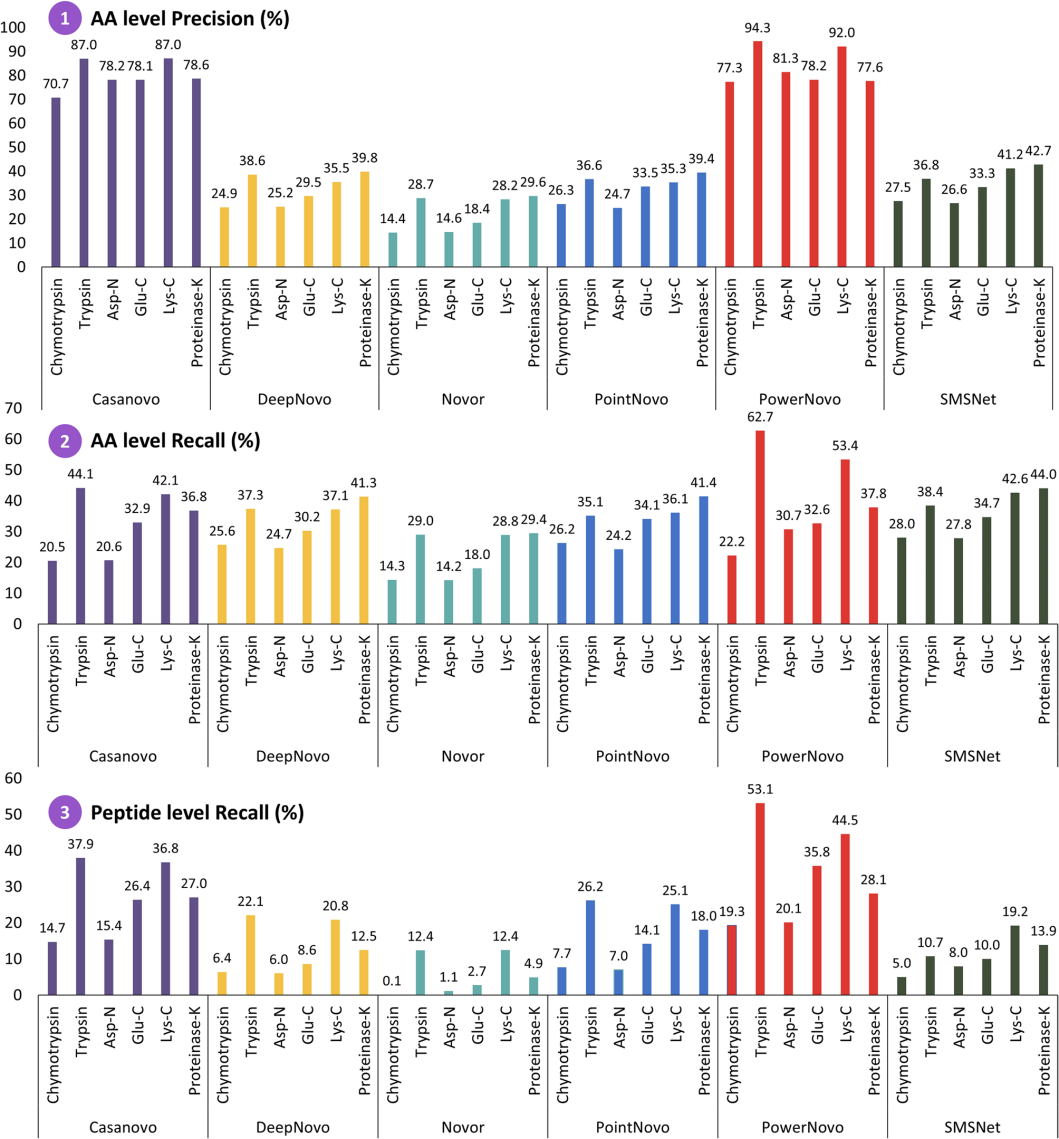
Total recall and precision of Novor, DeepNovo, SMSNet, PointNovo, Casanovo and PowerNovo across different enzymes on IgG1-Human-HC (dataset MSV000079801). (1) Precision at amino acid level. (2) Recall at amino acid level. (3) Recall at peptide level.

Hence, a conclusion can be drawn that PowerNovo ensures good precision at amino acid level and very high precision at the peptide level (Fig. 4).

For the remaining IgG1-Human-LC and Herceptin proteins, the respective diagrams are shown in Supplementary Figs. S7 and S8.

##### Analysis of typical errors

**Figure 5:**
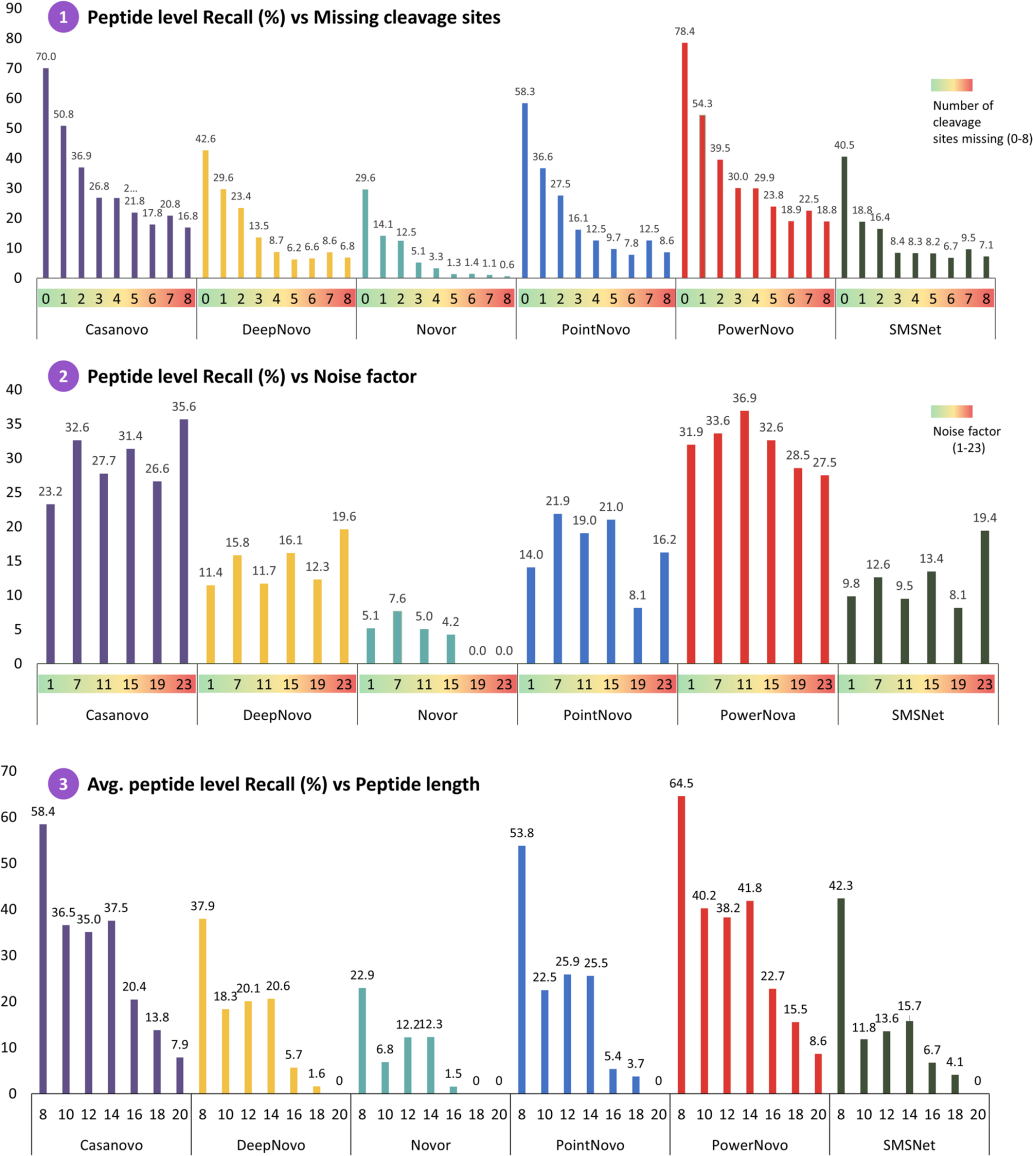
(1) Total peptide recalls of Novor, DeepNovo, SMSNet, PointNovo and PowerNovo across all data sets for different number of cleavage sites missing (datasets MSV000079801, PXD023419). (2) Total peptide recalls for different noise factors of the specific spectra. In the antibody dataset, each peak was labeled as a peptide peak or a noise peak. Peaks were labeled using the Pyteomics package, and noise peaks were labeled only if their intensity exceeded the median for the entire set. (3) Average recall at peptide level across peptide length.

One of the key problems of peptide sequencing algorithms is the presence of noise and missing fragment ions^9^. In our study, we evaluated the impact of these factors on the predictions made by our network and compared the outputs to those made by other algorithms. According to the description of the antibody dataset, 91% of all the spectra are missing at least one fragment ion, and ~ 84% of all peaks can be classified as noise peaks. Moreover, peptide sequence length significantly affects prediction precision (Fig. 5).

One can see from the outputs that prediction precision decreases significantly with increasing peptide length for all the algorithms without exception. The prediction precision also declines as the number of missed hydrolysis sites increases. These values are interrelated: long peptides usually have a large number of missed hydrolysis sites.

The Casanovo and PowerNovo tools ensure the highest precision. It is due to the implementation of transformer-based frameworks, which allow one to simultaneously process spectral peaks and study the relationships between amino acids using the self-attention mechanism. The use of the BERT model cutting off false sequences contributes to higher precision of the PowerNovo tool (Supplementary Fig. S5). Additionally, we identified and analyzed the key errors in amino acid sequence predictions (Supplementary Fig. S6).

According to the results of error analysis, a conclusion can be drawn that PowerNovo generated fewer predictions compared to other networks, but prediction precision was higher. The BERT block contributed to it as it cut off false hypotheses before they entered the post-processing algorithms. Noise in the spectrum did not significantly affect the precision of de novo sequencing algorithms (Fig. 5(2)).

##### Protein sequence assembly and protein-level performance evaluation

The de Bruijn ALPS graph assembler was used to test prediction accuracy of various de novo peptide sequencing tools at the protein level. This tool generates several intermediate sequences (contigs) based on the outputs of peptide sequence predictions. For PowerNovo, antibody sequences and contigs were aligned using a built-in algorithm; the BLAST software was used for the other tools. Table 2 lists the results of a comparative analysis of the top three outputs in terms of coverage. We used the top 20 contigs for each enzyme.

**Table 2:** Comparison of three de novo tools for protein sequence coverage.

	IgG LC (216 AA)	IgG HC (446 AA)	Herceptin LC (214 AA)	Herceptin HC (449 AA)
PointNovo				
Mapped contigs	34	50	14	21
Longest contig	42 (19.44%)	34 (7.62%)	36 (16.82%)	47 (10.47%)
Seq. coverage	180 (83.33%)	370 (82.96%)	156 (72.90%)	250 (55.68%)
Seq. accuracy	92%	93%	90%	90%
Casanovo				
Mapped contigs	6	12	5	7
Longest contig	24 (11.11%)	33 (7.40%)	34 (15.89%)	26 (5.79%)
Seq.coverage	58 (26.85%)	174 (39.01%)	85 (39.72%)	118 (26.28%)
Seq. accuracy	90%	95%	91%	92%
PowerNovo				
Mapped contigs	28	72	12	42
Longest contig	92 (42.59%)	101 (26.45%)	97 (45.33%)	108 (24.05%)
Seq. coverage	216 (100.00%)	446 (100%)	164 (76.64%)	412 (91.76%)
Seq. accuracy	92%	95%	97	93%

Contigs were constructed using de Bruijn assembler ALPS (k-mer = 8). The mapped contigs were aligned to the reference protein sequence using the BLAST algorithm. In this comparison, we only used contigs with at least 80% identity (BLAST Identity ≥ 0.8). Longest contig describes the maximum length of all generated contigs. Sequence coverage was calculated as the percentage of amino acids of the complete reference protein sequence covered by contigs. Accuracy was calculated as the percentage of all protein sequence calls that were labeled correctly. Full results (including the contigs) are shown in Supplementary Appendix S1.

We comprehensively evaluated performance of our PowerNovo solution, having compared it to the modern de novo algorithms, including PointNovo and Casanovo deep learning models. These models were selected because of their availability, matched network frameworks, and performance claimed in publications. Our study demonstrated that all of these algorithms can be successfully applied to solve peptide sequencing problems.

Using the BERT model as a tool for hypothesis evaluation yields more accurate and high-quality outputs. The PowerNovo solution meets the highest modern requirements; its performance is comparable or, in some cases, superior over that of other available de novo tools.

##### Performance evaluation of the BERT model

Performance of the BERT model was evaluated for prediction of the detectability of peptides according to their amino acid sequence in a test dataset (Homo sapiens from GPMDB) partially mixed with decoy sequences. Two recently published deep learning models, PepFormer^38^ and DeepMSPeptide^21^, were chosen for comparative analysis. Since the PepFormer and DeepMSPeptide models predict only peptide detectability, they were compared only for this task. Furthermore, we were also interested in how other models would behave on decoy sequences mixed into the dataset and what predictions would be generated for these sequences.

**Figure 6:**
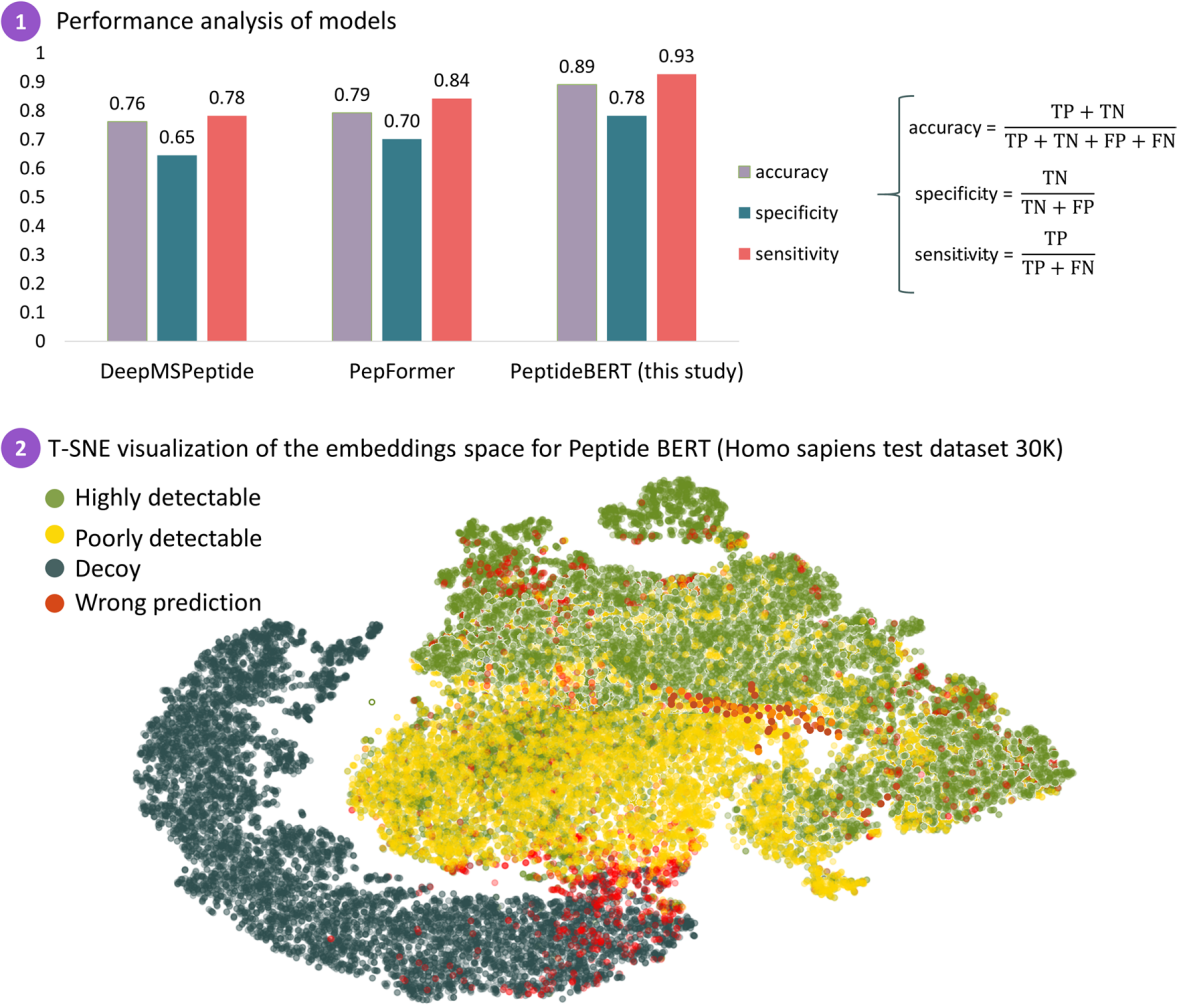
(1) Performance of DeepMSPeptide, PepFormer and Peptide BERT block (PowerNovo) for peptide detectability prediction with the test dataset from the GPMDB database (Homo sapiens (20 K) + decoys (10 K)). (2) T-SNE visualization of the latent embeddings space for Peptide BERT block. The visualization results indicate that our model can efficiently capture high-latent discriminative information, improving the predictive performance. Plot was obtained using python 3.9 script and matplotlib 3.8.0 library.

The study demonstrated (Fig. 6) that our model significantly outperforms other algorithms in this aspect. The analysis revealed that the DeepMSPeptide and PepFormer models classify some decoy sequences as easily detectable peptides, while our model does not. This fact confirms that the Peptide BERT model has great potential to reliably predict peptide detectability and can adapt to other types of organisms.

## Discussion

This paper presents a novel tool for de novo protein sequencing, which extends the boundaries of proteomic analysis for researchers. We discussed the new PowerNovo tool and analyzed its performance in antibody sequencing. The PowerNovo tool was also compared to other popular software packages such as Novor, DeepNovo, SMSNet, PointNovo, and Casanovo. We believe that our results show significant advances compared to the available de novo sequencing algorithms in proteomics.

The novelty of our solution lies in the fact that an auxiliary block based on the BERT framework and trained on a large peptide dataset has been added. It enables more accurate identification of the set of peptides present in the spectrum. Although this technique has been successfully applied in other domains such as speech technologies^39,40^, it has not been used before in the proteomics domain. One of the bottlenecks in developing artificial intelligence models in proteomics is that there exists no rigorous method for assessing the validity of de novo sequencing outputs. The Peptide BERT block trained on a large dataset in our solution helped us mitigate the evaluation-related uncertainty.

The disadvantage of our solution is the increase in computational time by 15–20% compared to the Casanovo tool, which also has a transformer-based structure. Another disadvantage is the tighter requirements for computing resources. Optimization of the algorithms is currently ongoing.

Along with general improvements compared to other modern de novo tools, we offer a comprehensive solution that involves peptide sequence assembly and mapping to proteins, while simultaneously solving the protein inference problem. This important additional feature will greatly simplify researchers’ work.

Furthermore, we expect that after our models are fine-tuned for performing specific tasks such as dealing with large datasets, individual post-translational modifications, and low-resolution spectra, they will learn to recognize novel natural or induced chemical modifications of peptide sequences, so their application in proteomics and for PTM detection and discovery will be broadened. This will contribute to further knowledge acquisition.

We hope that this study will be useful for the research community and promote the emergence of new ideas and solutions in artificial intelligence applications for processing and analyzing the experimental results in proteomics.

### Supplementary Information

Supplementary Information 1.

Supplementary Information 2.

## Data Availability

PowerNovo source code and trained model weights are available under the MIT license at https://github.com/protdb/PowerNovo. Datasets for training and validate models are available in the MassIVE repository with Library Name: KB 2.0.15, in vivo (https://massive.ucsd.edu/ProteoSAFe/static/massive-kb-libraries.jsp), dataset identifier MSV000081142, Peptide Atlas data with Library Name: THISP_2024-01-01 (https://db.systemsbiology.net/sbeams/cgi/PeptideAtlas/buildDetails?atlas_build_id=572) and GPMDB (https://gpmdb.thegpm.org/), NIST Libraries of Peptide Tandem Mass Spectra https://chemdata.nist.gov/dokuwiki/doku.php?id=peptidew:cdownload. The datasets analyzed during the current study are available in the MassIVE repository under registered ID: MSV000079801 and PRIDE repository under registered ID: PXD023419. Dataset files, result files and Python script to reproduce the results are available at Figshare (10.6084/m9.figshare.25378330).
