# Supplementary material for: PowerNovo: de novo peptide sequencing via tandem mass spectrometry using an ensemble of transformer and BERT models
Source: Sci Rep. 2024 Jul 1;14:15000. doi: 10.1038/s41598-024-65861-0 (PMC11217302; doi:10.1038/s41598-024-65861-0)
Supplement: Supplementary file 1 — Supplementary Information 1. [file 41598_2024_65861_MOESM1_ESM.docx]

# **Appendix**

**-----------CASANOVO-------------**

IG_HeavyChain: Mapped contigs: 12

IG_HeavyChain: Longest contig mapped: NKALPAPLEKTLSKAKGQPREPQVYTLPPSREE

IG_HeavyChain: Longest contig query: NKALPAPLEKTLSKAKGQPREPQVYTLPPSREE

IG_HeavyChain: Longest contig length: 33 (7.40%)

IG_HeavyChain: Coverage: 174 (39.01)%

IG_HeavyChain: Accuracy: 95.0

IG_LightChain: Mapped contigs: 6

IG_LightChain: Longest contig mapped: YACEVTHQGLSSPVTKSFNRGEC

IG_LightChain: Longest contig query: YACEVTHEGLSSPVTGKSFGRGGEC

IG_LightChain: Longest contig length: 24 (11.11%)

IG_LightChain: Coverage: 58 (26.85)%

IG_LightChain: Accuracy: 90.0

Heavychain_Herceptin: Mapped contigs: 7

Heavychain_Herceptin: Longest contig mapped: VVVDVSHEDPEVKFNWYVDGVEVHNA

Heavychain_Herceptin: Longest contig query: VVVDVSHEDPEVKFNWYVDGVEVHNA

Heavychain_Herceptin: Longest contig length: 26 (5.79%)

Heavychain_Herceptin: Coverage: 118 (26.28)%

Heavychain_Herceptin: Accuracy: 92.0

Lightchain_Herceptin: Mapped contigs: 5

Lightchain_Herceptin: Longest contig mapped: KVDNALQSGNSQESVTEQDSKDSTYSLSSTLTLS

Lightchain_Herceptin: Longest contig query: KVDNALQSGNSQESVTEQDSKDSTYSLSSTLTSL

Lightchain_Herceptin: Longest contig length: 34 (15.89%)

Lightchain_Herceptin: Coverage: 85 (39.72)%

Lightchain_Herceptin: Accuracy: 91.0

**-----------DEEPNOVO-------------**

IG_HeavyChain: Mapped contigs: 11

IG_HeavyChain: Longest contig mapped: MTSLDPVDTGTYYCAHTR

IG_HeavyChain: Longest contig query: MTSLDPVDTGTYYCAHTR

IG_HeavyChain: Longest contig length: 18 (4.04%)

IG_HeavyChain: Coverage: 120 (26.91)%

IG_HeavyChain: Accuracy: 96.0

IG_LightChain: Mapped contigs: 6

IG_LightChain: Longest contig mapped: VDNAIQSGNSQESVTEQDSK

IG_LightChain: Longest contig query: VDNALQSGNSQESVTEQDSK

IG_LightChain: Longest contig length: 20 (9.26%)

IG_LightChain: Coverage: 76 (35.19)%

IG_LightChain: Accuracy: 95.0

Heavychain_Herceptin: Mapped contigs: 13

Heavychain_Herceptin: Longest contig mapped: WGGDGFYAMDYWGQGTLVTVSSASTKGPSVFPLAPSSK

Heavychain_Herceptin: Longest contig query: WGGDGFYAMDYWGQGTLVTVSSASTKGPSVFPLAPSSK

Heavychain_Herceptin: Longest contig length: 38 (8.46%)

Heavychain_Herceptin: Coverage: 199 (44.32)%

Heavychain_Herceptin: Accuracy: 97.0

Lightchain_Herceptin: Mapped contigs: 6

Lightchain_Herceptin: Longest contig mapped: VQWKVDNALQSGNSQESVTEQDSKDSTY

Lightchain_Herceptin: Longest contig query: VQWKVDNALQSGNSQESVTEQDSKDSTY

Lightchain_Herceptin: Longest contig length: 28 (13.08%)

Lightchain_Herceptin: Coverage: 85 (39.72)%

Lightchain_Herceptin: Accuracy: 92.0

**-----------NOVOR-------------**

IG_HeavyChain: Mapped contigs: 23

IG_HeavyChain: Longest contig mapped: THTCPPCPAPELLGGPSVFLFPPKPK

IG_HeavyChain: Longest contig query: THTCYQMTPELLGGPSVFLFPPKPK

IG_HeavyChain: Longest contig length: 26 (5.83%)

IG_HeavyChain: Coverage: 212 (47.53)%

IG_HeavyChain: Accuracy: 90.0

IG_LightChain: Mapped contigs: 11

IG_LightChain: Longest contig mapped: ACEVTHQGLSSPVTKSFNRGEC

IG_LightChain: Longest contig query: ACQVTHQGLSSPVTKSFNGECR

IG_LightChain: Longest contig length: 23 (10.65%)

IG_LightChain: Coverage: 70 (32.41) %

IG_LightChain: Accuracy: 86.0

Heavychain_Herceptin: Mapped contigs: 14

Heavychain_Herceptin: Longest contig mapped: WGGDGFYAMDYWGQGTLVTVSSASTKGPSVFPLAPSSK

Heavychain_Herceptin: Longest contig query: WGGNGFYAMNYWGQGTLVTVSSASTKGSPVFPLAPSSK

Heavychain_Herceptin: Longest contig length: 38 (8.46%)

Heavychain_Herceptin: Coverage: 152 (33.85) %

Heavychain_Herceptin: Accuracy: 90.0

Lightchain_Herceptin: Mapped contigs: 17

Lightchain_Herceptin: Longest contig mapped: DNALQSGNSQESVTEQDSKDSTYSLSSTLTLSKADYEK

Lightchain_Herceptin: Longest contig query: VNALQSGNSQEWTQENSKNSTYSLSSTLTLSKADYEK

Lightchain_Herceptin: Longest contig length: 38 (17.76%)

Lightchain_Herceptin: Coverage: 102 (47.66) %

Lightchain_Herceptin: Accuracy: 88.0

**-----------POINTNOVO-------------**

IG_HeavyChain: Mapped contigs: 50

IG_HeavyChain: Longest contig mapped: VSNKALPAPLEKTLSKAKGQPREPQVYTLPPSRE

IG_HeavyChain: Longest contig query: VSNKALPAPLEKTLSKAKGQPREPQVYTLEFYQK

IG_HeavyChain: Longest contig length: 34 (7.62%)

IG_HeavyChain: Coverage: 370 (82.96)%

IG_HeavyChain: Accuracy: 93.0

IG_LightChain: Mapped contigs: 34

IG_LightChain: Longest contig mapped: DSTYSISSTLTLSKADYEKHKVYACEVTHQGLSSPVTKSFNR

IG_LightChain: Longest contig query: SDTYSLSSTLTLSKADYEKHKVYACEVTHQGLSSPVTKSFNR

IG_LightChain: Longest contig length: 42 (19.44%)

IG_LightChain: Coverage: 180 (83.33) %

IG_LightChain: Accuracy: 92.0

Heavychain_Herceptin: Mapped contigs: 21

Heavychain_Herceptin: Longest contig mapped: TCLVKGFYPSDIAVEWESNGQPENNYKTTPPVLDSDGSFFLYSKLTV

Heavychain_Herceptin: Longest contig query: GYGLKGFYPSDLAVEWESNGQPENNYKTTPPVLDSDGSFFLYSKLTV

Heavychain_Herceptin: Longest contig length: 47 (10.47%)

Heavychain_Herceptin: Coverage: 250 (55.68) %

Heavychain_Herceptin: Accuracy: 90.0

Lightchain_Herceptin: Mapped contigs: 14

Lightchain_Herceptin: Longest contig mapped: KVDNALQSGNSQESVTEQDSKDSTYSLSSTLTLSKA

Lightchain_Herceptin: Longest contig query: KVDNALQSGNSQESVTEQDSKDSTYSLSDTLTLSAK

Lightchain_Herceptin: Longest contig length: 36 (16.82%)

Lightchain_Herceptin: Coverage: 156 (72.90) %

Lightchain_Herceptin: Accuracy: 90.0

**-----------SMSNET-------------**

IG_HeavyChain: Mapped contigs: 59

IG_HeavyChain: Longest contig mapped: ALPAPLEKTLSKAKGQPREPQVYTLPPSREEM

IG_HeavyChain: Longest contig query: ALPAPLEKTLSKAKGQPERPQVYTLPPSSPTE

IG_HeavyChain: Longest contig length: 32 (7.17%)

IG_HeavyChain: Coverage: 338 (75.78) %

IG_HeavyChain: Accuracy: 89.0

IG_LightChain: Mapped contigs: 23

IG_LightChain: Longest contig mapped: QWKVDNAIQSGNSQESVTEQDSKDSTYSISSTLTLSKADYEKHKVYACEVTHQGLSSPVTKSFNRGEC

IG_LightChain: Longest contig query: NWKVDNALQSGDSKESVTEEDSKDSTYSLSSTLTLSKADYEKHKVYACEVTHEGLSSPVTKSFNNHNH

IG_LightChain: Longest contig length: 68 (31.48%)

IG_LightChain: Coverage: 167 (77.31) %

IG_LightChain: Accuracy: 88.0

Heavychain_Herceptin: Mapped contigs: 26

Heavychain_Herceptin: Longest contig mapped: LLGGPSVFLFPPKPKDTLMISRTPEVTCVVVDVSHEDPEVKFNWYVDG

Heavychain_Herceptin: Longest contig query: SLAAPSVFLFPPKPKDTLMLSRTPEVTAVVVDVSHEDPEVKFNWYVDG

Heavychain_Herceptin: Longest contig length: 48 (10.69%)

Heavychain_Herceptin: Coverage: 243 (54.12) %

Heavychain_Herceptin: Accuracy: 90.0

Lightchain_Herceptin: Mapped contigs: 13

Lightchain_Herceptin: Longest contig mapped: QWKVDNALQSGNSQESVTEQDSKDSTYSLSSTLTLSKADYEKHKVYACEVTHQGLSSPVTK

Lightchain_Herceptin: Longest contig query: NWKVDNALQSGNSQESVTEQDSKDSTYSLSSTLTLSKADYEKHKVYAAEVTHQGLSSPVTK

Lightchain_Herceptin: Longest contig length: 61 (28.50%)

Lightchain_Herceptin: Coverage: 171 (79.91) %

Lightchain_Herceptin: Accuracy: 91.0

**-----------POWERNOVO (without BERT) -------------**

IG_HeavyChain: Mapped contigs: 71

IG_HeavyChain: Longest contig mapped: SASTKGPSVFPLAPSSKSTSGGTAALGCLVKDYFPEPVTVSWNSGALTSGVHTFPAVLQSSGLYSLSSVVTVPSSSLGTQTYLCNVNHKPSNTKVDKRV

IG_HeavyChain: Longest contig query: GTSTKGPSVFPLAPSSKSTSGGTAALGCLVKDYFPEPVTVSWNSGALTSGVHTFPAVLQSSGLYSLSSVVTVPSSSLGTQTYLCNVNHKPSNTKVDKRG

IG_HeavyChain: Longest contig length: 99 (22.20%)

IG_HeavyChain: Coverage: 439 (98.43) %

IG_HeavyChain: Accuracy: 95.0

IG_LightChain: Mapped contigs: 25

IG_LightChain: Longest contig mapped: DEQLKSGTASVVCLLNNFYPREAKVQWKVDNAIQSGNSQESVTEQDSKDSTYSISSTLTLSKADYEKHKVYACEVTHQGLSSPVTKSFNRGE

IG_LightChain: Longest contig query: DEQLKSGTASVVCLLNNFYPREAKVQWKVDNALQSGNSQESVTEQDSKDSTYSLSSTLTLSKADYEKHKVYACEVTHGAGLSSPVTKSFNRGE

IG_LightChain: Longest contig length: 92 (42.59%)

IG_LightChain: Coverage: 216 (100.00) %

IG_LightChain: Accuracy: 92.0

Heavychain_Herceptin: Mapped contigs: 37

Heavychain_Herceptin: Longest contig mapped: SLSSVVTVPSSSLGTQTYICNVNHKPSNTKVDKKVEPKSCDKTHTCPPCPAPELLGGPSVFLFPPKPKDTLMISRTPEVTCVVVDVSHEDPEVKFNWYVDGVEVHNAKTKPREEQY

Heavychain_Herceptin: Longest contig query: SLSSVVTVPSSSLGTQTYLCDYVLHKPMTYTWVNHKPSNTKVDKKVEPKSCDKTHTCPPCPAPELLGGPSVFLFPPKPKDTLMLSRTPEVTCVVVDVSHEDPEVKFNWYVDGVEVHNAKTKPSGRGG

Heavychain_Herceptin: Longest contig length: 116 (25.84%)

Heavychain_Herceptin: Coverage: 412 (91.76)%

Heavychain_Herceptin: Accuracy: 92.0

Lightchain_Herceptin: Mapped contigs: 13

Lightchain_Herceptin: Longest contig mapped: DEQLKSGTASVVCLLNNFYPREAKVQWKVDNALQSGNSQESVTEQDSKDSTYSLSSTLTLSKADYEKHKVYACEVTHQGLSSPVTKSFNRGEC

Lightchain_Herceptin: Longest contig query: DEQLKSGTASVVCLLNNFYPREAKVQWKVDNALQSGNSQESVTEQDSKDSTYSLSSTLTLSKADYEKHKVYACEVTHQGLSSPVTKSFNRGEA

Lightchain_Herceptin: Longest contig length: 93 (43.46%)

Lightchain_Herceptin: Coverage: 172 (80.37) %

Lightchain_Herceptin: Accuracy: 93.0

**-----------POWERNOVO (with BERT) -------------**

IG_HeavyChain: Mapped contigs: 72

IG_HeavyChain: Longest contig mapped: AKGQPREPQVYTLPPSREEMTKNQVSLTCLVKGFYPSDLAVEWESNGQPENNYKTTPPVLDSDGSFFLYSKLTVDKSRWQQGNVFSCSVMHEALHNHYTQK

IG_HeavyChain: Longest contig query: AKGQPREPQVYTLPPSREEMTKNQVSLTCLVKGFYPSDLAVEWESNGQPENNYKTTPPVLDSDGSFFLYSKLTVDKSRWEQGGGVFSCSVMHEALHNHYTQK

IG_HeavyChain: Longest contig length: 101 (22.65%)

IG_HeavyChain: Coverage: 446 (100.00)%

IG_HeavyChain: Accuracy: 95.0

IG_LightChain: Mapped contigs: 28

IG_LightChain: Longest contig mapped: DEQLKSGTASVVCLLNNFYPREAKVQWKVDNAIQSGNSQESVTEQDSKDSTYSISSTLTLSKADYEKHKVYACEVTHQGLSSPVTKSFNRGE

IG_LightChain: Longest contig query: DEQLKSGTASVVCLLNNFYPREAKVQWKVDNALQSGNSQESVTEQDSKDSTYSLSSTLTLSKADYEKHKVYACEVTHGAGLSSPVTKSFNRGE

IG_LightChain: Longest contig length: 92 (42.59%)

IG_LightChain: Coverage: 216 (100.00)%

IG_LightChain: Accuracy: 92.0

Heavychain_Herceptin: Mapped contigs: 42

Heavychain_Herceptin: Longest contig mapped: SLSSVVTVPSSSLGTQTYICNVNHKPSNTKVDKKVEPKSCDKTHTCPPCPAPELLGGPSVFLFPPKPKDTLMISRTPEVTCVVVDVSHEDPEVKFNWYVDGVEVHNAK

Heavychain_Herceptin: Longest contig query: KDKKVEPKSCDKTHTCPPCPAPELLGGPSVFLFPPKPKDTLMLSRTPEVTCVVVDVSHEDPEVKFNWYVDGVEVHNAKTKPREEQYNSTYRSVFERDRDDSNGVLLTVLHQ

Heavychain_Herceptin: Longest contig length: 108 (24.05%)

Heavychain_Herceptin: Coverage: 412 (91.76)%

Heavychain_Herceptin: Accuracy: 93.0

Lightchain_Herceptin: Mapped contigs: 12

Lightchain_Herceptin: Longest contig mapped: IFPPSDEQLKSGTASVVCLLNNFYPREAKVQWKVDNALQSGNSQESVTEQDSKDSTYSLSSTLTLSKADYEKHKVYACEVTHQGLSSPVTKSFNRGE

Lightchain_Herceptin: Longest contig query: LFPPSDEQLKSGTASVVCLLNNFYPREAKVQWKVDNALQSGNSQESVTEQDSKDSTYSLSSTLTLSKADYEKHKVYACEVTHQGLSSPVTKSFNRGE

Lightchain_Herceptin: Longest contig length: 97 (45.33%)

Lightchain_Herceptin: Coverage: 164 (76.64)%

Lightchain_Herceptin: Accuracy: 96.0
