# Supplementary material for: PowerNovo: de novo peptide sequencing via tandem mass spectrometry using an ensemble of transformer and BERT models
Source: Sci Rep. 2024 Jul 1;14:15000. doi: 10.1038/s41598-024-65861-0 (PMC11217302; doi:10.1038/s41598-024-65861-0)
Supplement: Supplementary file 2 — Supplementary Information 2. [file 41598_2024_65861_MOESM2_ESM.docx]

**Supplementary**

## Training datasets

**Table S1.** Description of the datasets for training the transformer model.

| SOURCE | SPECIES | Method of fragmentation | Data acquisition technique | PEPTIDES | PRECURSORS | DESCRIPTION | Link |
| --- | --- | --- | --- | --- | --- | --- | --- |
| Massive-KB  Spectral library v2 - full releases  KB 2.0.15, in vivo | H.sapiens | HCD | DDA | [1,958,922](https://massive.ucsd.edu/ProteoSAFe/result.jsp?task=e33a302ea7e94422bf2b122260d22cc6&view=peptides_per_dataset) | [4,843,818](https://massive.ucsd.edu/ProteoSAFe/result.jsp?task=e33a302ea7e94422bf2b122260d22cc6&view=ambiguity_library_view_split) | Human peptide spectral library constructed from 1.2 billion spectra from in vivo proteomics experiments (Orbitrap Fusion), including spectra from multiple data types.  The dataset consists of three parts:  - non-tryptic peptides (PSMs 720,167) | https://massive.ucsd.edu/ProteoSAFe/static/massive-kb-libraries.jsp |
| NIST | H.sapiens | CID ^41^ | DDA | 207,910 | 340,356 | Human Ion Trap Library (low-resolution MS/MS from several different instruments).  To evaluate the performance of the model, the dataset was split into a training set and a test set. 10,000 samples were used for testing. | https://chemdata.nist.gov/dokuwiki/doku.php?id=peptidew:lib:human20140529 |
| NIST | H.sapiens | HCD | DDA | 605,677 | 911,783 | Consensus Human HCD Libraries.  The dataset consists of three parts^42^:  - high-quality spectra, mostly tryptic peptides without missed cleavages (PSMs 3421);  - medium-quality spectra, mostly tryptic peptides with missed cleavages (PSMs 805);  - high- and medium-quality spectra, mostly semi-tryptic peptides (PSMs 850).  To evaluate the performance of the model, the dataset was split into a training set and a test set. 10,000 samples from each part were used for testing. | https://chemdata.nist.gov/dokuwiki/doku.php?id=peptidew:lib:humanhcd20160503 |
| NIST | H.sapiens | HCD | DDA/DIA | 390,009 | 1,201,632 | The human HCD iTRAQ library was created entirely from raw data stored on the Clinical Proteomic Tumor Analysis Consortium (CPTAC) data portal.  The dataset consists of the parts^43^:  - phosphoproteome (55 Studies, PSMs 14,074);  - glycoproteome (10 Studies, PSMs 342);  - acetylome (12 Studies, PSMs 902);  - proteome (71 Studies, PSMs 23,814).  DDA (137 Studies, PSMs 59,244)  DIA (12 Studies) | https://chemdata.nist.gov/dokuwiki/doku.php?id=peptidew:clib:humanhcditraqselected20141126 |
| NIST  PXD004732 | H.sapiens | ETD | DDA | 188,805 | 696,692 | Human Synthetic Peptide Spectral Library (Orbitrap Fusion ETD).  The dataset consists of the parts^43^: | https://chemdata.nist.gov/dokuwiki/doku.php?id=peptidew:lib:human_hair_selected_with_gvps_passed |
| NIST | M.musculus | CID | DDA | 149,458 | 94,055 | Mouse Ion Trap Library To evaluate the performance of the model, the data set was split into a training set and a validation set. 10,000 samples were used for testing. | https://chemdata.nist.gov/dokuwiki/doku.php?id=peptidew:lib:mouse20130520 |
| NIST | E. coli | CID | DDA | 62,383 | 36,230 | E Coli Ion trap library.  To evaluate the performance of the model, the data set was split into a training set and a test set. 10,000 samples were used for testing. | https://chemdata.nist.gov/dokuwiki/doku.php?id=peptidew:lib:e_coli |
| NIST | Yeast  (S.cerevesiae) | CID | DDA | 92,609 | 92,609 | Yeast Ion Trap Library. To evaluate the performance of the model, the data set was split into a training set and a validation set. 10,000 samples were used for testing. | https://chemdata.nist.gov/dokuwiki/doku.php?id=peptidew:lib:yeast_it |
| NIST | Yeast  (S.cerevesiae) | CID | DDA | 14,647 | 12,657 | Yeast Collision Cell (QTOF) Library. To evaluate the performance of the model, the data set was split into a training set and a test set. 1,000 samples were used for testing. | https://chemdata.nist.gov/dokuwiki/doku.php?id=peptidew:lib:yeast_qtof |
| Massive-KB  MSV000081142 | H.sapiens | HCD | DDA | [1,087,062](https://massive.ucsd.edu/ProteoSAFe/result.jsp?task=f9beeb4d4c9a4d9ca45f141e8eda6597&view=view_result_list) | [2,121,033](https://massive.ucsd.edu/ProteoSAFe/result.jsp?task=f9beeb4d4c9a4d9ca45f141e8eda6597&view=view_result_list) | The validation set is used during the training phase of the model (Q Exactive). | https://massive.ucsd.edu/ProteoSAFe/dataset.jsp?task=f9beeb4d4c9a4d9ca45f141e8eda6597 |

**Table S2.** Description of the test datasets.

| SOURCE | SPECIES | PEPTIDES | PRECURSORS | DESCRIPTION | Link |
| --- | --- | --- | --- | --- | --- |
| NIST | H.sapiens | 10,000 | 10,000 | Human Ion Trap Library.  10,000 samples were used for testing. | https://chemdata.nist.gov/dokuwiki/doku.php?id=peptidew:lib:human20140529 |
| NIST | H.sapiens | 30, 000 | 30, 000 | Consensus Human HCD Libraries.  The dataset consists of three parts:   1. high-quality spectra, mostly tryptic peptides without missed cleavages; 2. medium-quality spectra, mostly tryptic peptides with missed cleavages; 3. high- and medium-quality spectra, mostly semi-tryptic peptides.   10,000 samples from each part were used for testing. | https://chemdata.nist.gov/dokuwiki/doku.php?id=peptidew:lib:humanhcd20160503 |
| NIST | H.sapiens  (hair) | 6,280 | 2,240 | Human Hair Peptide Spectral Library (Fusion Lumos – HCD) | https://chemdata.nist.gov/dokuwiki/doku.php?id=peptidew:lib:human_hair_selected_with_gvps_passed |
| NIST | H.sapiens | 10, 000 | 10, 000 | Human Phosphopeptide Spectral Library (Orbitrap -HCD). 10, 000 samples were used for testing. | https://chemdata.nist.gov/dokuwiki/doku.php?id=peptidew:lib:human_hair_selected_with_gvps_passed |
| Massive-KB MassIVE-KB v2.0.15: Nontryptic only | H.sapiens | 10, 000 | 10, 000 | MassIVE-KB v2, MS/MS spectra of peptides from proteomics experiments digested with various different enzymes. 10, 000 samples were used for testing. | https://massive.ucsd.edu/ProteoSAFe/static/massive-kb-libraries.jsp |
| NIST | M.musculus | 17,851 | 10,026 | Mouse HCD Library. | https://chemdata.nist.gov/dokuwiki/doku.php?id=peptidew:clib:mousehcd_selected20141124 |
| NIST | M.musculus | 10,000 | 10,000 | Mouse Ion Trap Library. 10, 000 samples were used for testing. | https://chemdata.nist.gov/dokuwiki/doku.php?id=peptidew:lib:mouse20130520 |
| NIST | E. coli | 10,000 | 10,000 | E Coli Ion trap library.  10, 000 samples were used for testing. | https://chemdata.nist.gov/dokuwiki/doku.php?id=peptidew:lib:e_coli |
| NIST | Yeast  (S.cerevesiae) | 10,000 | 10,000 | Yeast Ion Trap Library. 10,000 samles were used for testing. | https://chemdata.nist.gov/dokuwiki/doku.php?id=peptidew:lib:yeast_it |
| NIST | Yeast  (S.cerevesiae) | 1, 000 | 1, 000 | Yeast Collision Cell (QTOF) Library. 1,000 samples were used for testing. | https://chemdata.nist.gov/dokuwiki/doku.php?id=peptidew:lib:yeast_qtof |
| NIST | Yeast  (Pombe) | 10,000 | 10,000 | Yeast Pombe Ion Trap Library. 10,000 samples were used for testing. | https://chemdata.nist.gov/dokuwiki/doku.php?id=peptidew:lib:yeastpombe |

**Table S3.** Description of the datasets for training the peptide BERT model.

| SOURCE | SEQUENCES | LABEL | DESCRIPTION |
| --- | --- | --- | --- |
| Massive-KB, GPMDB | 163,053 | 2 | Highly detectable peptides |
| Massive-KB, GPMDB | 4,268,960 | 1 | Poorly detectable peptides (potentially detectable) |
| Decoys from Human all proteome, THISP_2022-10-01 | 16,267,014 | 0 | Decoy |

## **Overview of model architecture**


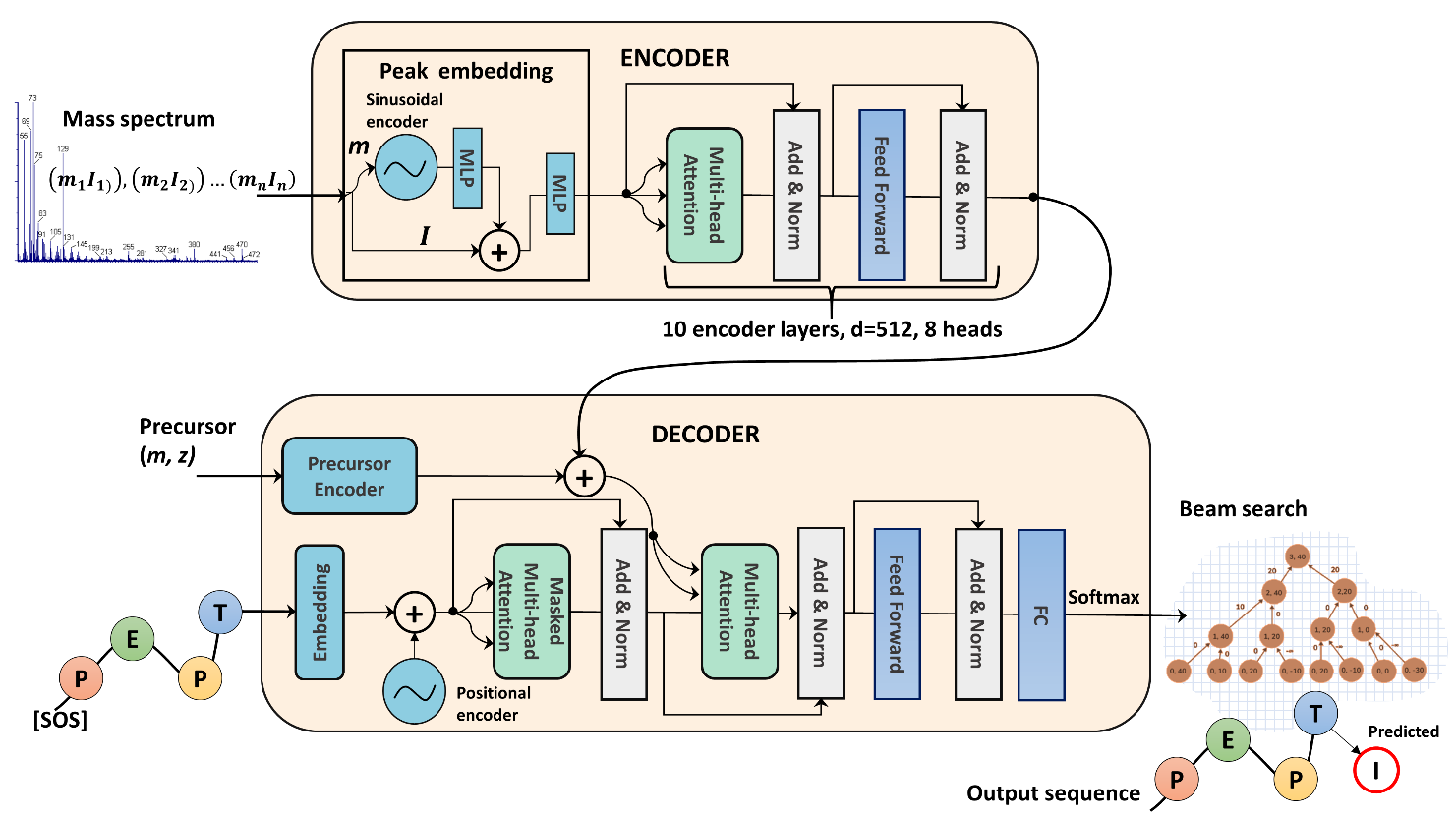


**Figure S1.** Details of the transformer model architecture.

#
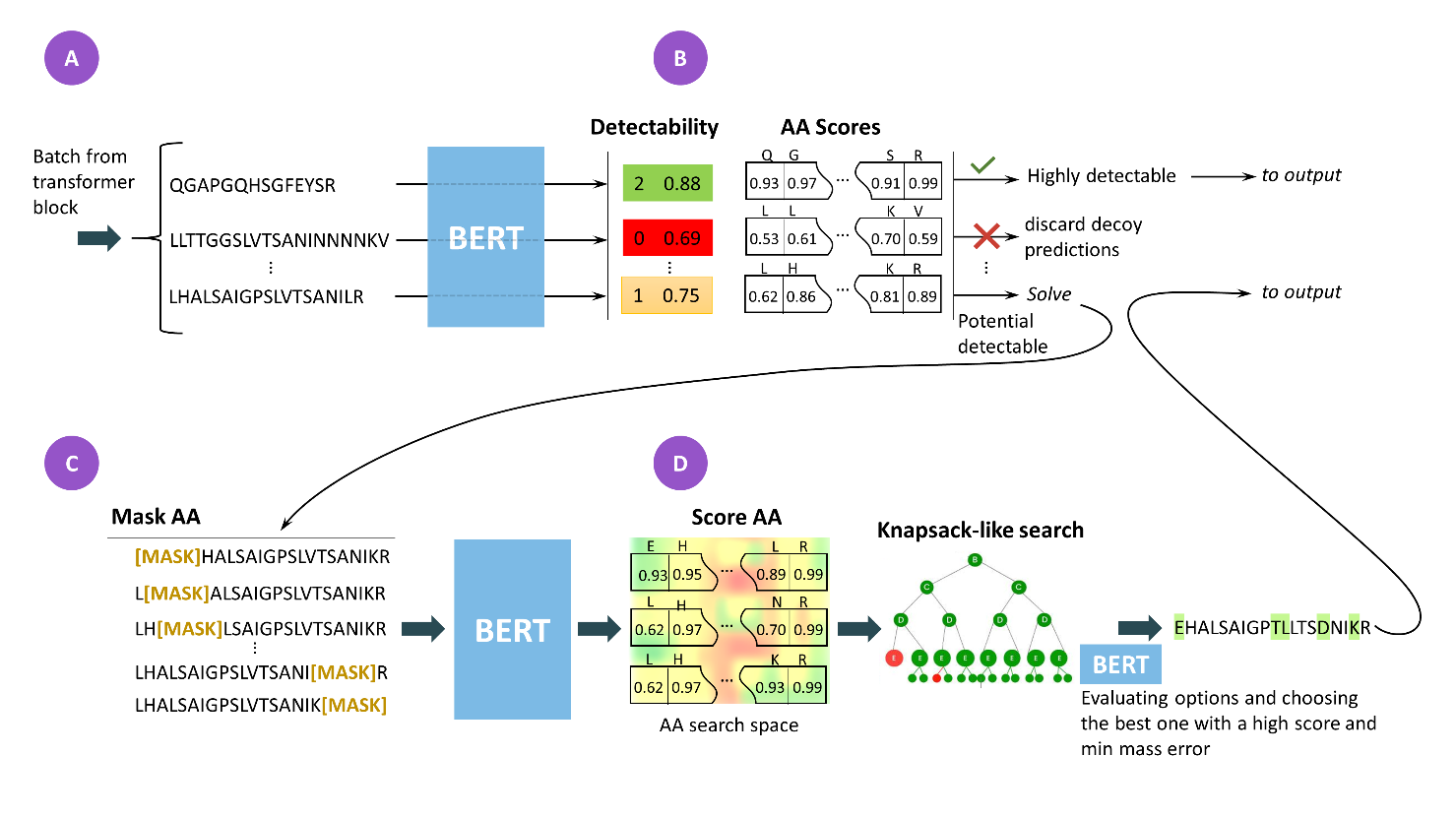
**Figure S2.** Basic principles for evaluating hypotheses with the BERT model: a) The input to the model is the peptide sequences predicted by the transformer model. b) The detectability of the sequences is assessed. Decoy sequences are excluded from further consideration. Sequences with a high detectability score are accepted as good. Sequences with low detectability scores are further analyzed by algorithms (c) and (d). c) The amino acids of the sequences are masked and passed through the BERT model, the output of which is a probability distribution of amino acid residues. This distribution forms the search space for possible options. d) Possible solutions are generated from the search space using the knapsack algorithm. A solution is selected that has a minimum mass error and a maximum detectability score.

## Description of antibody datasets and parameters for running de novo algorithms

**Table S4.** An overview of evaluated antibody datasets.

| Description | Database ID | MS | Ionization | Resolution | Enzymes | Reference |
| --- | --- | --- | --- | --- | --- | --- |
| IgG1-Human | MSV000079801 | LTQ Orbitrap | HCD | 17,500 | Trypsin, chymotrypsin, asp-N, lys-C, glu-C, proteinase K | Tran NH, Rahman MZ, He L, et al. Complete de novo assembly of monoclonal antibody sequences. Sci Rep 2016;6(1):31730. |
| Herceptin | PXD023419 | Orbitrap Fusion | Stepped HCD and EThcD | 30,000 | Trypsin, thermolysin, lys-N, lys-C, glu-C, asp-N, aLP, chymotrypsin, elastase | Peng W, Pronker MF, Snijder J. Mass spectrometry-based de novo sequencing of monoclonal antibodies using multiple proteases and a dual fragmentation scheme. J Proteome Res 2021;20(7):3559–66. |

In order to evaluate the de novo sequencing algorithms, we compared each peptide sequence prediction to pseudo-ground truth data taken from the results reported by D. Beslic et al. (2023). Comprehensive evaluation of de novo peptide sequencing tools for monoclonal antibody assembly. Briefings in bioinformatics, 24(1), bbac542. https://doi.org/10.1093/bib/bbac542). It was needed because the datasets presented in Table S4 contain untokened spectra. According to this paper, tokening of the amino acid sequence in the spectra was performed by searching across the databases containing sequences of these antibodies using the MS-GF+ and X!Tandem tools. All the peptide spectrum matches were filtered out under 1% FDR.

**Table S5.** Parameters for de novo software packages.

| Parameters | Value | Comments |
| --- | --- | --- |
| Precursor tolerance | 10 ppm |  |
| Fragment mass tolerance | 0.02 Da |  |
| Fixed modifications | Cysteine (C + 57.02 Da) |  |
| Variable modifications | - oxidation of methionine (M + 15.99 Da); - deamidation of asparagine (N + 0.98 Da); - and deamidation of glutamine (G + 0.98 Da) | PowerNovо also supports phospho-PTMs (S + 79.97 Da), (T + 79.97 Da), (Y + 79.97 Da), PowerNovo also supports the output of phosphorylated PTMs, but for consistency we excluded these from the analysis |

The ALPS assembler takes the de novo confidence score into consideration for the assembly, but it could generate incorrect results with a large amount of low-confidence k-mers. The authors of DeepNovo recommend removing sequence contaminants from de novo sequencing results by excluding peptides with a confidence score below 50 to improve the quality of the assembly.

## Evaluation


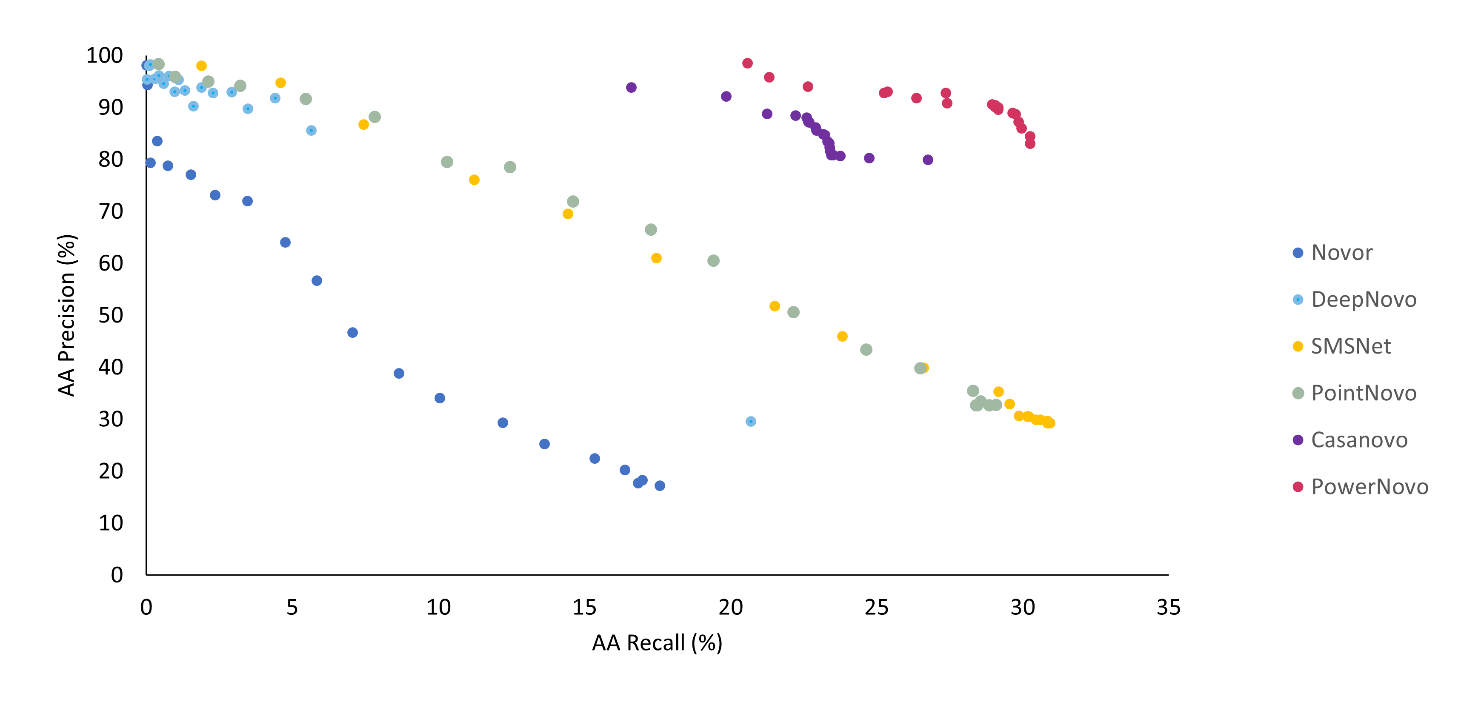


**Figure S3.** The PR curves of Novor, DeepNovo, SMSNet, PointNovo, Casanovo and PowerNovo tools. The average recall and precision for all the enzymes of IgG1-Human-HC.

**Table S6** Comparison of PowerNovo, CasaNovo and DeepNovo (peptide-level).

The table lists the peptide-level precision and recall of three models on the test dataset (Table S2).

| Species | Type | Description | Peptide-level performance | | | | | |
| --- | --- | --- | --- | --- | --- | --- | --- | --- |
|  |  |  | DeepNovo | | Casanovo | | PowerNovo | |
|  |  |  | Prec. | Recall | Prec. | Recall | Prec. | Recall |
| H.sapiens | Ion Trap | Human Ion Trap Library. | 0.18 | 0.18 | 0.24 | 0.18 | 0.27 | 0.23 |
| H.sapiens | HCD | High-quality spectra. (mostly tryptic peptides w/o missed cleavages). | 0.39 | 0.39 | 0.61 | 0.50 | 0.74 | 0.71 |
| H.sapiens | HCD | Medium-quality spectra (mostly peptides with missed cleavages). | 0.33 | 0.33 | 0.55 | 0.43 | 0.69 | 0.62 |
| H.sapiens | HCD | High- and medium-quality spectra, mostly semi-tryptic peptides. | 0.36 | 0.36 | 0.58 | 0.49 | 0.76 | 0.72 |
| H.sapiens | HCD | Human Hair Peptide (Fusion Lumos – HCD). | 0.11 | 0.11 | 0.12 | 0.08 | 0.02 | 0.02 |
| H.sapiens | HCD | Nontryptic only peptides. | 0.24 | 0.25 | 0.39 | 0.3 | 0.48 | 0.42 |
| M.musculus | HCD | Mouse HCD Library. | 0.43 | 0.43 | 0.60 | 0.51 | 0.67 | 0.62 |
| M.musculus | Ion Trap | Mouse Ion Trap Library. | 0.25 | 0.25 | 0.31 | 0.22 | 0.33 | 0.25 |
| E. coli | Ion Trap | E Coli Ion Trap library. | 0.20 | 0.20 | 0.30 | 0.22 | 0.29 | 0.26 |
| Yeast  (S.cerevesiae) | Ion Trap | Yeast Ion Trap Library. | 0.27 | 0.27 | 0.32 | 0.27 | 0.34 | 0.3 |
| Yeast  (S.cerevesiae) | Collision Cell (QTOF) | Yeast Collision Cell (QTOF) Library. | 0.14 | 0.14 | 0.18 | 0.15 | 0.17 | 0.15 |
| Yeast  (Pombe) | Ion Trap | Yeast Pombe Ion Trap Library. |  |  |  |  |  |  |

**Table S7** Comparison of PowerNovo, CasaNovo and DeepNovo (amino acid-level).

The table lists the peptide-level precision and recall of three models on the test dataset (Table S2).

| Species | Type | Description | AA-level performance | | | | | |
| --- | --- | --- | --- | --- | --- | --- | --- | --- |
|  |  |  | DeepNovo | | Casanovo | | PowerNovo | |
|  |  |  | Prec. | Recall | Prec. | Recall | Prec. | Recall |
| H.sapiens | Ion Trap | Human Ion Trap Library. | 0.35 | 0.35 | 0.39 | 0.37 | 0.43 | 0.41 |
| H.sapiens | HCD | High-quality spectra. (mostly tryptic peptides w/o missed cleavages). | 0.63 | 0.60 | 0.75 | 0.7 | 0.91 | 0.87 |
| H.sapiens | HCD | Medium-quality spectra (mostly peptides with missed cleavages). | 0.56 | 0.53 | 0.66 | 0.64 | 0.83 | 0.80 |
| H.sapiens | HCD | High- and medium-quality spectra, mostly semi-tryptic peptides. | 0.59 | 0.57 | 0.75 | 0.69 | 0.89 | 0.86 |
| H.sapiens | HCD | Human Hair Peptide (Fusion Lumos – HCD). | 0.20 | 0.16 | 0.16 | 0.13 | 0.11 | 0.09 |
| H.sapiens | HCD | Nontryptic only peptides. | 0.47 | 0.45 | 0.55 | 0.5 | 0.65 | 0.6 |
| M.musculus | HCD | Mouse HCD Library. | 0.66 | 0.65 | 0.74 | 0.7 | 0.82 | 0.8 |
| M.musculus | Ion Trap | Mouse Ion Trap Library. | 0.43 | 0.4 | 0.40 | 0.38 | 0.43 | 0.41 |
| E. coli | Ion Trap | E Coli Ion Trap library. | 0.40 | 0.38 | 0.42 | 0.40 | 0.46 | 0.44 |
| Yeast  (S.cerevesiae) | Ion Trap | Yeast Ion Trap Library. | 0.47 | 0.44 | 0.48 | 0.46 | 0.52 | 0.48 |
| Yeast  (S.cerevesiae) | Collision Cell (QTOF) | Yeast Collision Cell (QTOF) Library. | 0.31 | 0.30 | 0.46 | 0.45 | 0.46 | 0.48 |
| Yeast  (Pombe) | Ion Trap | Yeast Pombe Ion Trap Library. | 0.26 | 0.26 | 0.40 | 0.38 | 0.41 | 0.39 |


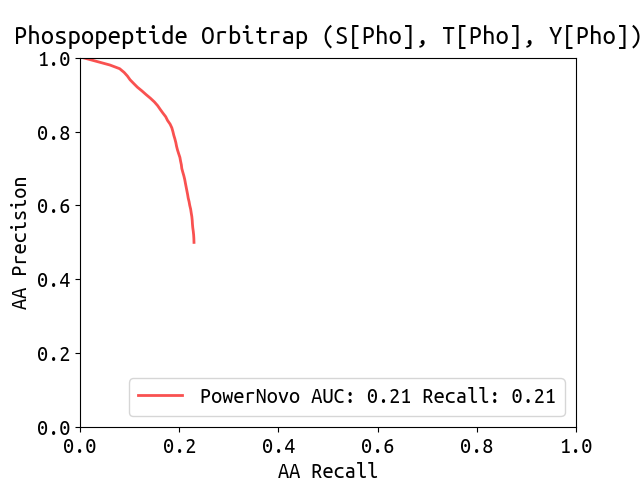


**Figure S4.** Amino acid-level precision and recall of three post-translational modifications (Phosphoserine, O-Phosphotyrosine, Phosphothreonine). The NIST Human Phosphopeptide Spectral Library (Orbitrap -HCD) was used for analysis.


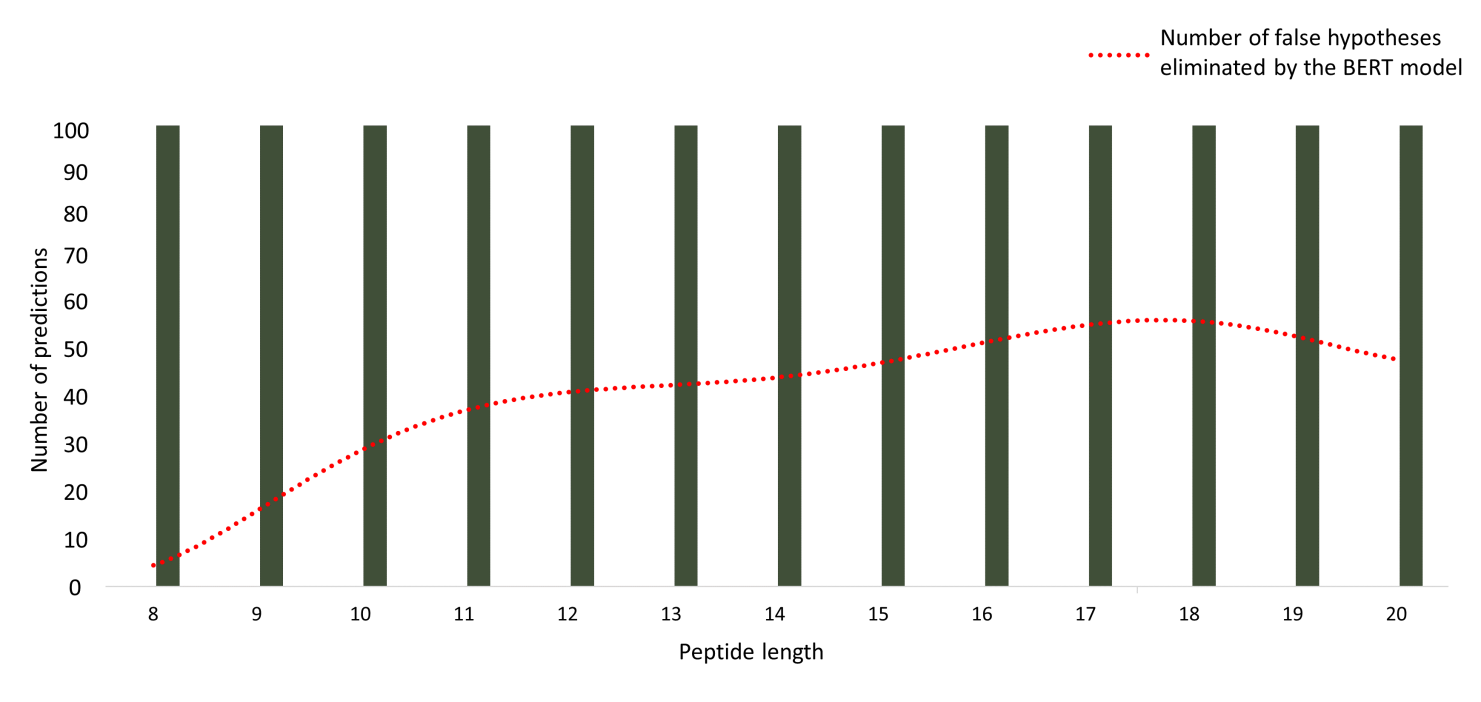


**Figure S5.** The number of false hypotheses (highly noisy or decoy peptide sequences) cut off by the BERT model depending on peptide length.


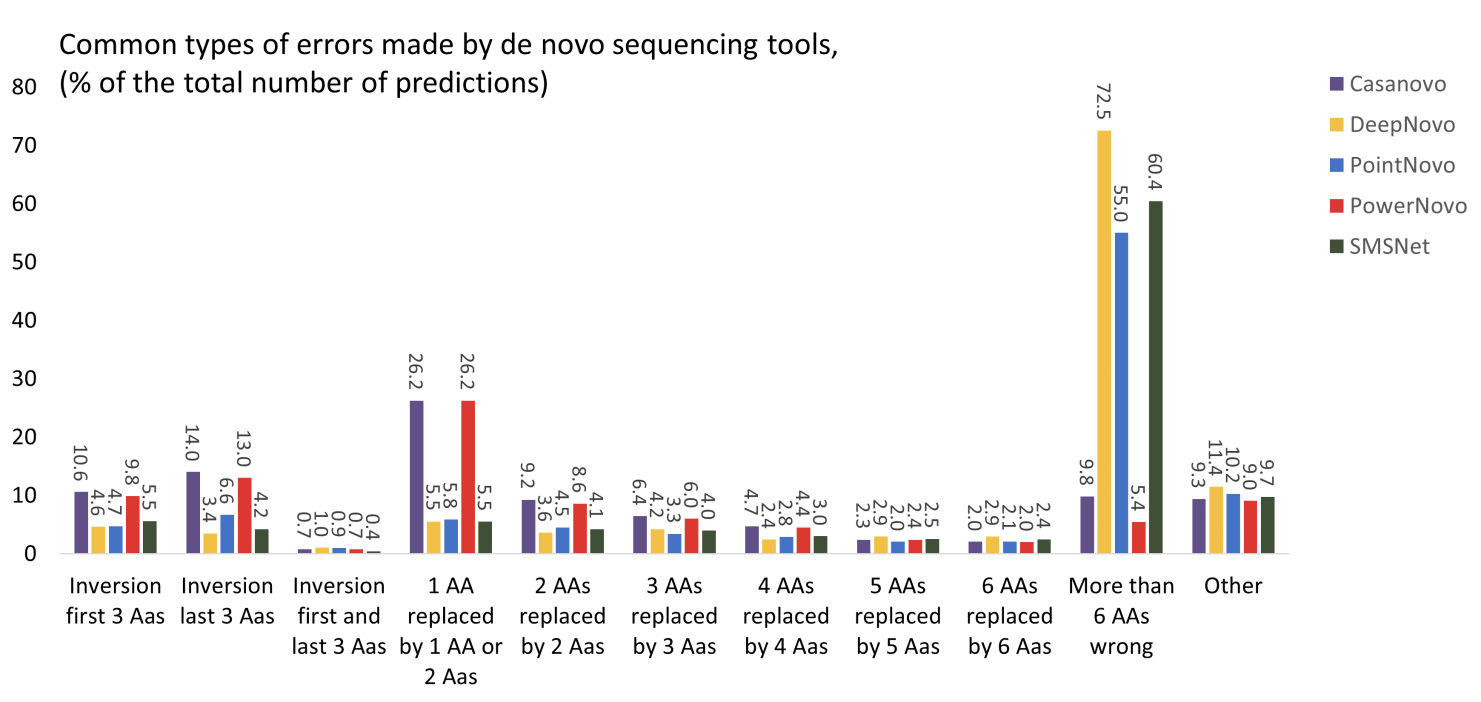


**Figure S6.** Types of errors made by de novo sequencing algorithm tools on the IgG1-Human and Herceptin datasets (datasets MSV000079801, PXD023419). The percentage of errors to the total number of predictions is shown. Errors are mapped into the 10 most common error categories. The “Other” category includes errors that do not fall into any other category.


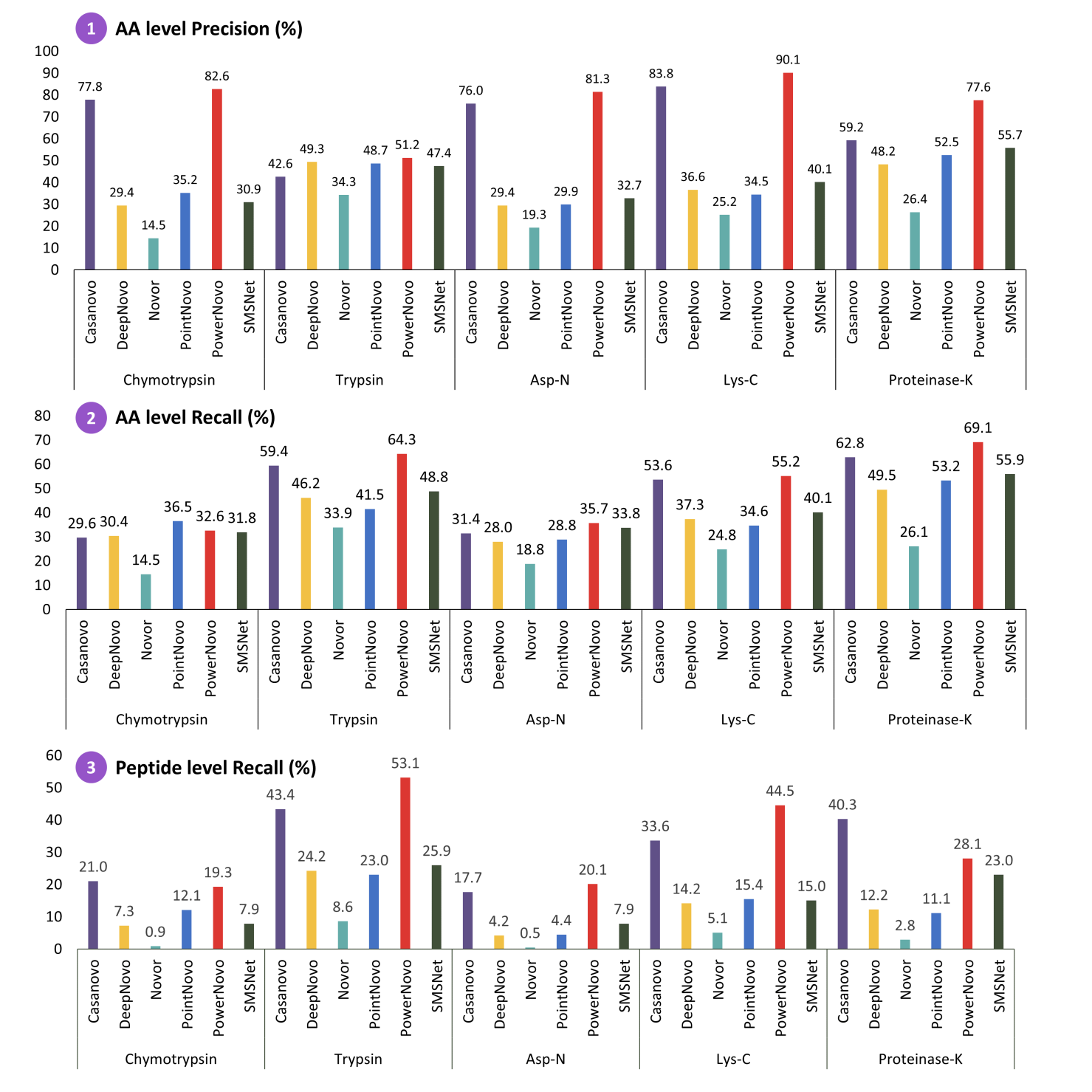


**Figure S7.** Total recall and precision of Novor, DeepNovo, SMSNet, PointNovo, Casanovo and PowerNovo tools across different enzymes on IgG1-Human-LC (dataset MSV000079801). (1) Precision at amino acid level. (2) Recall at amino acid level. (3) Recall at peptide level.


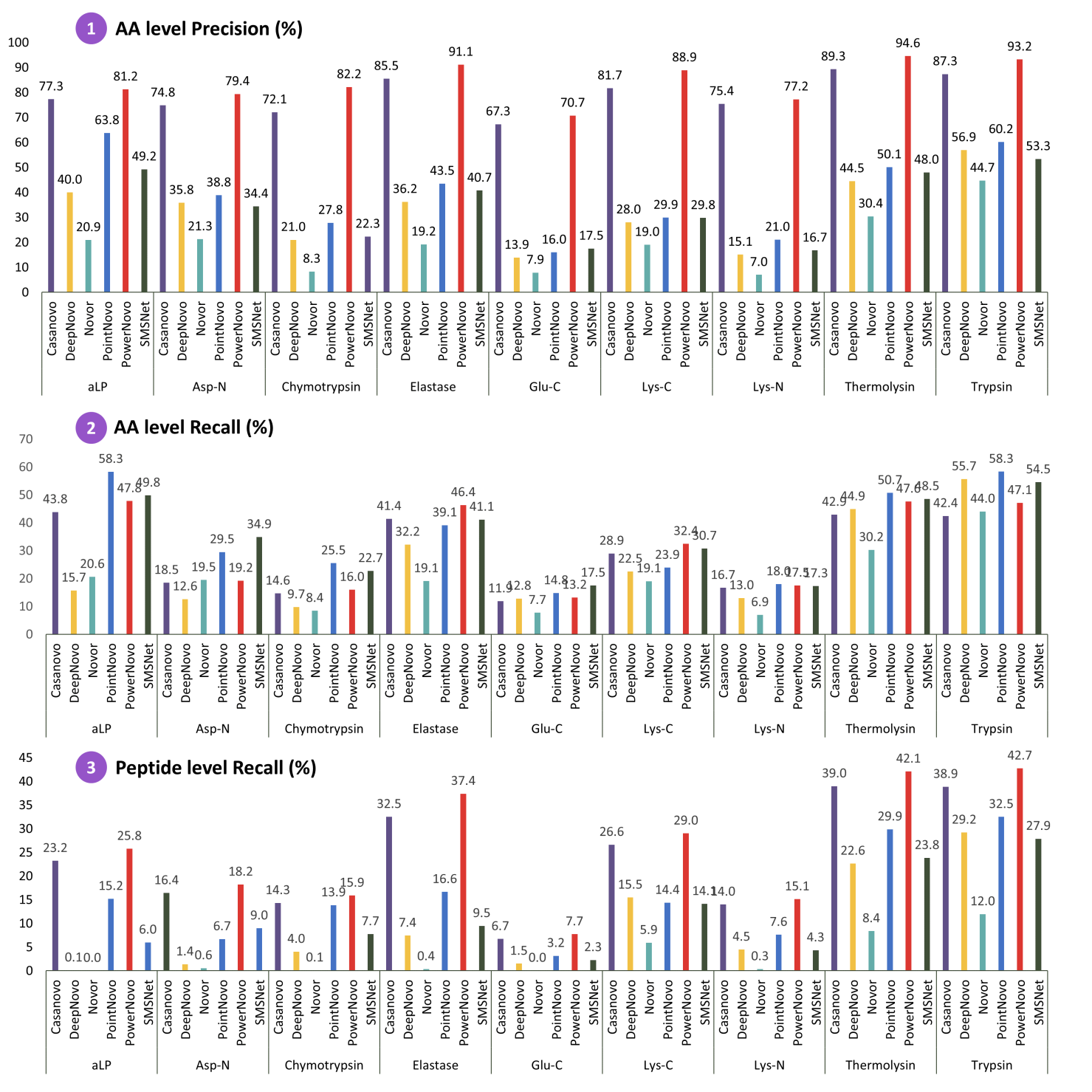


**Figure S8.** Total recall and precision of Novor, DeepNovo, SMSNet, PointNovo, Casanovo and PowerNovo tools across different enzymes on Herceptin (dataset PXD023419). (1) Precision at amino acid level. (2) Recall at amino acid level. (3) Recall at peptide level.
